# TOP2A Maintains Heterochromatin Stability by Sustaining *LMNB1* Expression to Counteract Senescence

**DOI:** 10.1111/acel.70787

**Published:** 2026-11-02

**Authors:** Ke Cheng, Yaru Wang, Yueyuan Pan, Wenjun Zhang, Denghong Yu, Qi Zhou, Zhiqian Zhong, Ruyi Mei, Chaoliang Xu, Ayidana Hayierhan, Yumei Ma, Yien Xu, Qingdong Xie, Pingnan Sun, Junzhi Zhou, Xiaoling Zhou

**Affiliations:** ^1^ Stem Cell Research Center Shantou University Medical College Shantou China; ^2^ The Center for Reproductive Medicine Shantou University Medical College Shantou China; ^3^ Department of Gynecology First Affiliated Hospital, Shantou University Medical College Shantou China; ^4^ School of Basic Medicine, Guangdong Provincial Key Laboratory of Medical Immunology and Molecular Diagnostics Guangdong Medical University Dongguan China

**Keywords:** aging, heterochromatin, LMNB1, TOP2A

## Abstract

Chromatin stability and nuclear architectural integrity constitute the primary defense against cellular senescence. Their collapse precipitates genomic disarray, epigenetic misregulation, and functional decline in stem cells. Although TOP2A is conventionally known for resolving DNA topological stress, its role in aging remains largely unexplored. Here, we found that TOP2A expression progressively decreased in senescent mesenchymal stem cells (MSCs), and its depletion initiated a catastrophic nuclear remodeling process characterized by the loss of Lamin B1, heterochromatin erosion, and senescence‐associated phenotypes. Mechanistically, TOP2A directly regulated the expression of Lamin B1 to maintain nuclear architecture. TOP2A depletion set off a domino effect involving nuclear membrane rupture, loss of heterochromatin‐associated factors (H3K9me3, KAP1, and LAP2), deterioration of cellular functions and acceleration of senescence. Notably, overexpression of TOP2A restored Lamin B1 levels, mitigated nuclear protein loss, preserved nuclear integrity, and counteracted cellular senescence. The in vivo relevance of these findings was further underscored by TOP2A inhibitor‐induced brain aging in murine models. Our study establishes TOP2A as a novel modulator of cellular senescence, which stabilizes heterochromatin and nuclear structure, and provides new perspectives for targeting chromatin‐based mechanisms in aging.

## Graphical abstract

Depletion of TOP2A accelerates MSC senescence and disrupts heterochromatin integrity. TOP2A directly regulates *LMNB1* expression to maintain MSC nuclear integrity and epigenomic stability. Overexpression of TOP2A counteracts MSC senescence through Lamin B1‐mediated chromatin reorganization and epigenomic stabilization. Pharmacological inhibition of TOP2A triggers premature brain aging and neurodegenerative‐like behavioral phenotypes in mice.

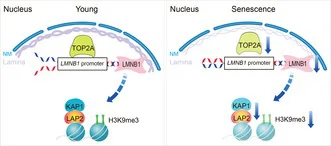

## 1 Introduction

Adult stem cell exhaustion is a hallmark of organismal aging (Guo et al. 2022; Lopez‐Otin et al. 2013, 2023). MSCs are multipotent stromal cells derived from the mesoderm, and are characterized by their high capacity for self‐renewal and multi‐lineage differentiation. These cells can differentiate into various cell types, including osteoblasts, chondrocytes, and adipocytes. Aged MSCs exhibit diminished regenerative capacity, contributing to tissue dysfunction and systemic decline (Deng et al. 2021; Fei et al. 2014; Geyh et al. 2013; Li et al. 2017; Malaise et al. 2019; Plakhova et al. 2023; Xie et al. 2019; Ye et al. 2023). Thus, mitigating MSC senescence will prove beneficial for reprogramming aging processes and serve as an intervention strategy for aging‐related diseases (Gao et al. 2023; Hu et al. 2022; Kim et al. 2021). MSC senescence involves epigenetic dysregulation, such as heterochromatin loss, nuclear membrane disruption, and mitochondrial dysfunction, leading to genomic instability and senescence‐associated secretory phenotypes (SASP) (Cheung et al. 2018; Pal and Tyler 2016; Sen et al. 2016; Song et al. 2022; Wu et al. 2024). Notably, heterochromatin stabilization is emerging as a key strategy to counteract cellular senescence (Della Valle et al. 2022; L. Deng et al. 2019; Zhang, Liu, et al. 2021). However, the upstream regulators linking nuclear architecture to MSC senescence remain poorly defined.

DNA topoisomerases are essential enzymes that resolve topological constraints during chromatin dynamics, transcription, replication, and DNA repair, thereby safeguarding genome integrity (Champoux 2001; Pommier et al. 2022; Pommier et al. 2016; Vos et al. 2011; J. C. Wang 2002). Among them, topoisomerase II (Topo II) is an important member that has the ability to temporarily cleave and relegate double‐stranded DNA. This enzyme facilitates the untangling of DNA strands, thereby addressing topological challenges that arise from modifications in the helical structure of DNA (Nitiss 2009; Uuskula‐Reimand and Wilson 2022). TOP2A, which belongs to the Topo II family, is also critical in maintaining cell proliferation, sustaining stemness potential (Ben‐Moshe et al. 2022; Hishida et al. 2022; Qin et al. 2022), and is involved in embryonic development (Thakurela et al. 2013). In TOP2A knockout mouse models, the inability to complete chromosome segregation leads to embryonic lethality (Akimitsu et al. 2003; Duan et al. 2022). In addition, TOP2A is also involved in adult neurogenesis (Qin et al. 2022), neurodevelopmental disorders, and inherited retinal diseases (Ledvin et al. 2023; Roy et al. 2022; Watanabe et al. 1994). Heterochromatin stability is essential for adult stem cells to resist aging, although it remains unclear whether TOP2A plays a significant role in this process. Elucidating the underlying molecular mechanisms is of great significance for developing novel anti‐aging strategies.

Here, we demonstrate that TOP2A downregulation is both a marker and driver of MSC senescence. Mechanistically, TOP2A directly stabilizes Lamin B1, preventing loss of nuclear membrane and heterochromatin‐associated proteins, thereby preserving nuclear integrity. Conversely, TOP2A downregulation accelerates senescence via nuclear dysmorphia, while its overexpression rescues these defects by restoring Lamin B1 expression and mitigating the loss of nuclear membrane and heterochromatin‐associated proteins. In vivo, TOP2A inhibition induces brain aging in mice, highlighting its systemic relevance. Overall, our findings reveal TOP2A as a functional node in adult stem cell aging, bridging genome topology and nuclear stability and leading to novel strategies to combat aging by TOP2A targeting.

## 2 Results

### 2.1 TOP2A Expression Is Decreased in Senescent Human MSCs

In a core factor analysis related to MSC senescence, we identified 328 upregulated and 586 downregulated genes (|log_2_FC| ≥ 1) associated with senescence through analysis of RNA‐seq data from MSCs of different donors and passages (GSE35957 and GSE30391, comprising 11 young and 11 senescent samples). Heatmap analysis revealed that proliferation markers ( *MKI67* , *PCNA* , and *MCM2* ) were downregulated while senescence markers ( *CDKN1A* and *CDKN2A* ) were upregulated in the senescence group, validating the senescent phenotype. The most significantly altered genes included upregulated (e.g., *RPS4Y1* , *MYOZ2* ) and downregulated (e.g., *TOP2A* , *TTK* ) transcripts (Figure 1A,B). Applying stringent criteria (|log_2_FC| > 3, adj. *p* < 0.05), we derived a senescence‐related gene set A (26 genes). Weighted gene co‐expression network analysis (WGCNA) of the same data yielded a module of 83 senescence‐associated genes (set B) (Figure S1A), including core aging regulatory gene ( *CDKN2A* ), cell cycle regulatory genes (*CCNB1, FBXO5, CDKN3*), DNA damage repair and genome stability‐related genes (*TOP2A, RAD51, BARD1, FANCI and HMGB2*), and anti‐apoptotic and proliferation marker genes (*BIRC5 and MKI67*). Additionally, differential expression analysis of young ( *CDKN1A* ^*−*^ *MKI67* ^+^) and senescent (*CDKN1A* ^+^ *MKI67* ^*−*^) MSCs from a single‐cell dataset (https://data.mendeley.com/datasets/f4b2ykfv56/1) (Figure S1B,C) provided a 10‐gene set C, containing *TOP2A* , *CCNB1* , and *HMGB2* , among others. Intersection of the three gene sets revealed TOP2A as a core senescence‐associated factor (Figure 1C). This DNA topoisomerase, critical for genomic stability, is downregulated in senescent and late‐passage MSCs, and could be one of the core factors closely related to MSC senescence.

**FIGURE 1:**
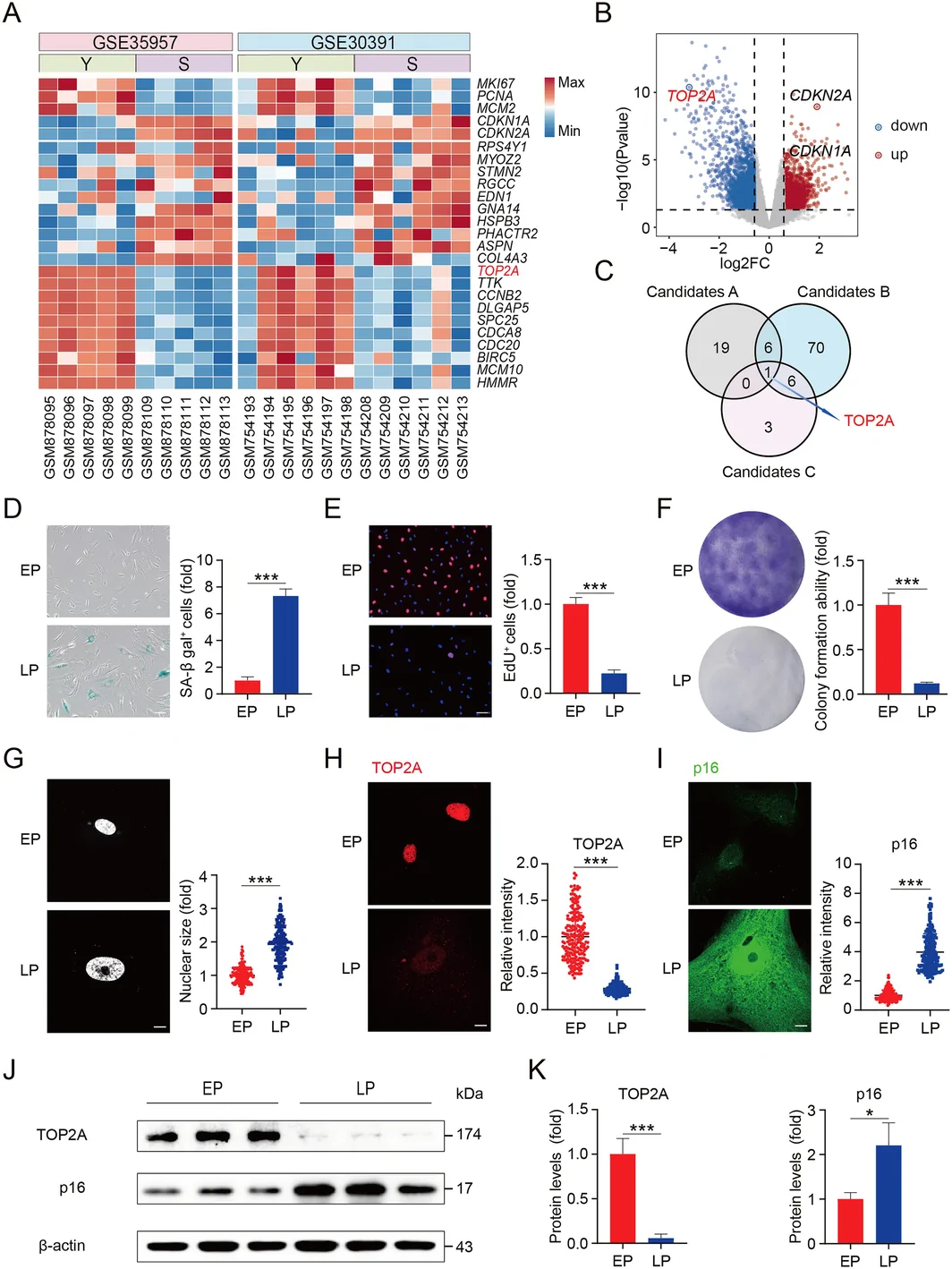
TOP2A expression is decreased in senescent human MSCs. (A) Heatmap showing the top 10 differentially‐expressed genes (DEGs) between young and senescent MSCs (Y: Young, S: Senescent). (B) Volcano plot of DEGs between young and senescent MSCs. (C) Venn diagram showing the intersection of candidate senescence‐related genes in sets A, B, and C. (D) SA‐β‐gal staining (blue) of MSCs at early passage (EP) and late passage (LP). Scale bar: 50 μm. *N* = 5 biological repeats, *p* < 0.001. (E) EdU incorporation assay quantifying proliferating cells (red) in MSCs at early passage and late passage. Scale bar: 50 μm. *N* = 4 biological repeats, *p* < 0.001. (F) Colony formation assays assessing clonogenic potential of MSCs at early passage and late passage. *N* = 3 biological repeats, *p* < 0.001. (G) Nuclear area quantification of MSCs at early passage and late passage. Scale bar: 10 μm. *N* = 200 cells per group, *p* < 0.001. (H) Immunofluorescence staining of TOP2A (red) in MSCs. Scale bar: 10 μm. *N* = 200 cells per group, *p* < 0.001. (I) Immunofluorescence analysis of p16 (green) in MSCs. Scale bar: 10 μm. *N* = 200 cells per group, *p* < 0.001. (J, K) Western blot analysis of TOP2A and p16 expression in replicative senescent MSCs. *N* = 3 biological repeats, TOP2A (*p* < 0.001), p16 (*p* = 0.015). Early passage (EP) is defined as passage 4 (P4). Late passage (LP) is defined as P13. Statistical significance was determined by the independent samples *t*‐test. Data are presented as the mean ± SD. *p < 0.05, ***p < 0.001. Source data are available online for this figure.

To examine whether the expression of TOP2A decreases during adult stem cell aging, we isolated MSCs from human umbilical cord to construct replicative‐senescent MSCs (Figure S1D–F). Early‐passage MSCs displayed typical morphology: small cells with defined outlines, minimal granularity, and intact nuclei. Late‐passage cells, in contrast, exhibited classic senescence features: enlarged, flattened morphology with increased granularity and fragmented nuclei (He et al. 2024; Heckenbach et al. 2022). Consistent with this, SA‐β‐gal staining revealed significantly more senescent cells in late‐passage cultures (Figure 1D). EdU staining confirmed a marked reduction in proliferating (S‐phase) late‐passage cells (Figure 1E). Furthermore, late‐passage MSCs showed a significant decrease in colony‐forming ability (Figure 1F). Nuclear enlargement and flattening of senescent cells (Belhadj et al. 2023; Heckenbach et al. 2022; Lomakin et al. 2020), which were quantified by nuclear size, were assessed via immunofocusing in late passage MSCs (Figure 1G). We also found that the fluorescence intensity of p16 was up‐regulated while TOP2A was down‐regulated in late passage MSCs (Figure 1H,I), which was consistent with the senescence phenotype characteristics (Childs et al. 2017; Lopez‐Otin et al. 2023). In addition, the level of p16 protein expression was stably up‐regulated in late‐generation MSCs, while that of TOP2A was stably down‐regulated (Figure 1J,K).

Further, we validated the downregulation of TOP2A in senescent MSCs isolated from different donor origins (Figure S2A–F). We also identified that *TOP2A* is downregulated in senescent MSCs in different tissues (GSE98440, GSE160273) (Yu et al. 2023; Zirkel et al. 2018; Figure S3A,B). Additionally, our analysis revealed a downregulation of *TOP2A* expression across multiple organs including lung, spleen, bladder, stomach, liver, and intestine, with increasing human age in the ADEIP Human Multi‐Organ Aging Public Database (Liu et al. 2021; Figure S3C). Collectively, TOP2A‐downregulation exhibits a consistent expression pattern during aging, and may serve as a potential biomarker in senescent human MSCs. Furthermore, its conserved reduction strongly suggests a functional role as a critical regulator in MSC senescence.

### 2.2 Depletion of TOP2A Accelerates Cellular Senescence

To further dissect the role of TOP2A in MSC aging, we sought to determine whether the inhibition of TOP2A expression would induce senescence in MSCs. To investigate this, we first employed TOP2A siRNA to treat the MSCs and confirmed cellular senescence by SA‐β‐gal staining and a colony formation assay (Figure S4A,B). Subsequently, we used a TOP2A specific inhibitor, PluriSIn #2 (TOP2Ai), to selectively repress TOP2A transcription (Ben‐David et al. 2015; Qin et al. 2022). We used TOP2Ai to treat MSCs and examined the expression of TOP2A and TOP2B. We found that *TOP2A* expression was down‐regulated and *CDKN1A* expression was up‐regulated, while *TOP2B* remained unchanged (Figure S4C). Therefore, we used the TOP2Ai to treat MSCs in our experiments. As expected, similar to TOP2A siRNA, TOP2Ai treatment led to the senescence of MSCs, with many more SA‐β‐gal‐positive cells in the TOP2Ai‐treated MSCs than in the DMSO control group (Figure 2A). In a clonal formation assay, cell colonies in the TOP2Ai group were fewer than the DMSO control group, indicating that the clonogenic ability of TOP2Ai‐treated MSCs decreased, consistent with induction of senescence (Figure 2B). Also, the nuclei in TOP2Ai‐treated MSCs were enlarged (Figure 2C). In a fluorescence confocal experiment, the fluorescence intensity of typical senescent markers p21 and p16 was increased while TOP2A fluorescence was decreased after TOP2Ai treatment of MSCs (Figure 2D,E). We also examined TOP2A and classical senescence markers by western blotting, and the expression of TOP2A was down‐regulated when p21 and p16 were up‐regulated in the TOP2Ai‐treated MSCs (Figure 2F).

**FIGURE 2:**
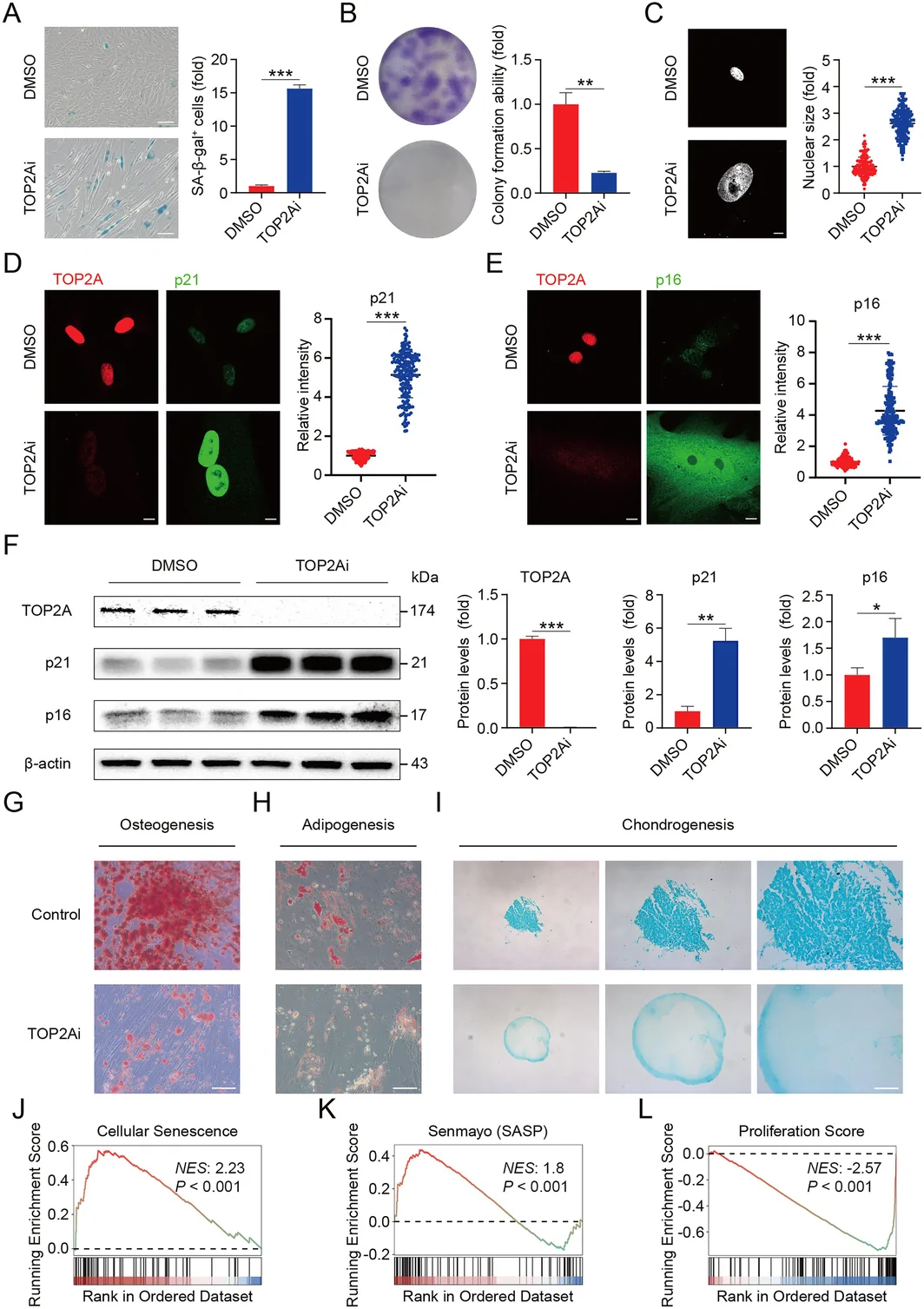
Depletion of TOP2A accelerates cellular senescence. (A) SA‐β‐gal staining showing senescent cells (blue). Scale bar: 100 μm. *N* = 5 biological repeats, *p* < 0.001. (B) MSC colony formation. *N* = 3 independent experiments, *p* = 0.009. (C) Quantification of nuclear area enlargement. Scale bar: 10 μm. *N* = 200 cells per group, *p* < 0.001. (D) Immunofluorescence staining of p21. Scale bar: 10 μm. *N* = 200 cells per group, *p* < 0.001. (E) Immunofluorescence staining of p16. Scale bar: 10 μm. *N* = 200 cells per group, *p* < 0.001. (F) Western blot analysis of TOP2A, p21, and p16 protein levels (β‐Actin loading control). *N* = 3 independent experiments, TOP2A (*p* < 0.001), p21 (*p* = 0.005), p16 (*p* = 0.034). (G) Osteogenic differentiation assay (Alizarin Red S staining). Scale bar: 100 μm. (H) Adipogenic differentiation assay (Oil Red O staining). Scale bar: 100 μm. (I) Chondrogenic differentiation assay (Alcian blue staining). Scale bar: 100 μm. (J) GSEA showing upregulation of the cellular senescence pathway. (K) GSEA showing upregulation of the senescence‐associated secretory phenotype (SASP) pathway. (L) GSEA demonstrating downregulation of cell proliferation pathways. Statistical analysis was performed using the independent samples *t*‐test. Data are presented as the mean ± SD. *p < 0.05, **p < 0.01, ***p < 0.001. Source data are available online for this figure.

MSC senescence can lose the differentiation capacity of MSCs into osteoblasts, adipoblasts, and chondrocytes (Huang et al. 2017; Park et al. 2005; Voskamp et al. 2023; Yang et al. 2018; Yu et al. 2024). As expected, the trilineage differentiation of MSCs was impaired, following inhibition of TOP2A by TOP2Ai (Figure 2G–I and Figure S4D). GSEA analysis of transcriptome sequencing data from MSCs treated with TOP2Ai revealed up‐regulation of pathways associated with cellular senescence (Figure 2J) and the senescence‐associated secretory phenotype (SASP) (Saul et al. 2022; Figure 2K). Conversely, pathways related to cell proliferation were downregulated (Figure 2L). SASP is a hallmark of senescent cells and is characterized by the secretion of various pro‐inflammatory factors, chemokines, and proteases. This phenomenon plays a crucial role in cellular senescence and has a substantial impact on the surrounding tissue microenvironment (Basisty et al. 2020; Saul et al. 2022; Takahashi et al. 2018; Wang et al. 2024). Therefore, we further examined classical SASP‐related genes in TOP2A‐downregulated MSCs, including *IL6* , *IL1A* , *IL1B* , *MMP3* , *AREG* , *ERE*G, *PAPPA* , *SERPINE1* , and *SERPINE2* (Andriani et al. 2016; Basisty et al. 2020; Conover and Bale 2023; Laberge et al. 2021; Taylor et al. 2024; von Joest et al. 2022; Wang et al. 2022; Wei et al. 2023; Xu et al. 2019), and found that they were all up‐regulated (Figure S4E,F).

Cellular senescence is associated with the downregulation of various cellular functions including mitochondrial function, proliferation, and cell cycle arrest. Previous studies have found that TOP2 exists in the form of a TOP2‐DNA complex in the D‐loop region of mtDNA in human and murine mitochondria, specifically at positions flanking the 7S DNA (Hangas et al. 2018). Our GSEA analysis revealed down‐regulation of the mitochondrial gene maintenance pathway in the inhibitor‐treated group (Figure S4G). Consequently, we assessed the mitochondrial membrane potential of MSCs subjected to TOP2Ai treatment (Figure S4H). Treatment with TOP2Ai led to a reduction in the mitochondrial membrane potential of MSCs. Additionally, EdU staining of MSCs indicated a significant reduction in the number of cells incorporating EdU following treatment with TOP2Ai, suggesting a marked decrease in DNA synthesis within this cohort (Figure S4I). Furthermore, the ability of the cells to proliferate was adversely affected. It has been posited that TOP2A expression is dependent on the cell cycle, with peak levels occurring during G2/M phase (Duan et al. 2022). TOP2A dysfunction activates cell cycle checkpoints, resulting in G2 phase or mid‐G1 phase arrest depending on the cell types (Deiss et al. 2019; Downes et al. 1994; Duan et al. 2022; Lopez et al. 2025; Zhang et al. 2025). Consequently, we sought to investigate whether TOP2Ai would similarly induce cell cycle checkpoint activation and thereby alter the functional characteristics of MSCs. Downregulation of TOP2A in MSCs resulted in ‐an increased proportion  of cells arresting in G1 phase (Figure S4J,K). Overall, these results indicate that downregulation of TOP2A accelerates MSC senescence and impairs cellular function.

### 2.3 Depletion of TOP2A Leads to Disruption of Heterochromatin Integrity

Abnormalities in heterochromatin are one of the important hallmarks of epigenetic aging (Amoiridis et al. 2024; Xie et al. 2024; Zhang et al. 2015). Nuclear remodeling in senescent cells, driven by histone post‐translational modifications and DNA methylation shifts, induces global heterochromatin loss during senescence (Cruickshanks et al. 2013; De Cecco et al. 2013; Suryadevara et al. 2024). Previous studies have found that TOP2A is essential for the maintenance of chromosome structure and that TOP2A depletion leads to loss of mitotic chromosome structure (Nielsen et al. 2020). Likewise, we investigated whether the downregulation of TOP2A causes the loss of heterochromatin‐associated proteins within the nucleus. Notably, GSEA analysis of our transcriptome sequencing data revealed downregulation of heterochromatin formation and organization (Figure 3A,B). Clustering heat map analysis also revealed that heterochromatin‐associated genes were down‐regulated in the TOP2Ai group (Figure 3C). Expression of heterochromatin‐associated histone marks and proteins, specifically H3K9me3 (Nicetto et al. 2019; Zhang et al. 2024), nuclear lamina proteins (LAP2) (Gant and Wilson 1997; Li et al. 2024), KRAB‐associated protein 1 (KAP1) (Bi et al. 2020; Diao et al. 2021a) were down‐regulated in the TOP2Ai group (Figure 3D,E). Consistent with the results of western blotting, the fluorescence intensities of KAP1, LAP2, H3K9me3, and heterochromatin protein 1 homolog alpha (HP1α) in TOP2Ai‐treated MSCs were decreased (Figure 3F–I). Taken together, our results suggest that depletion of TOP2A leads to loss of heterochromatin proteins and thereby impairs integrity of heterochromatin.

**FIGURE 3:**
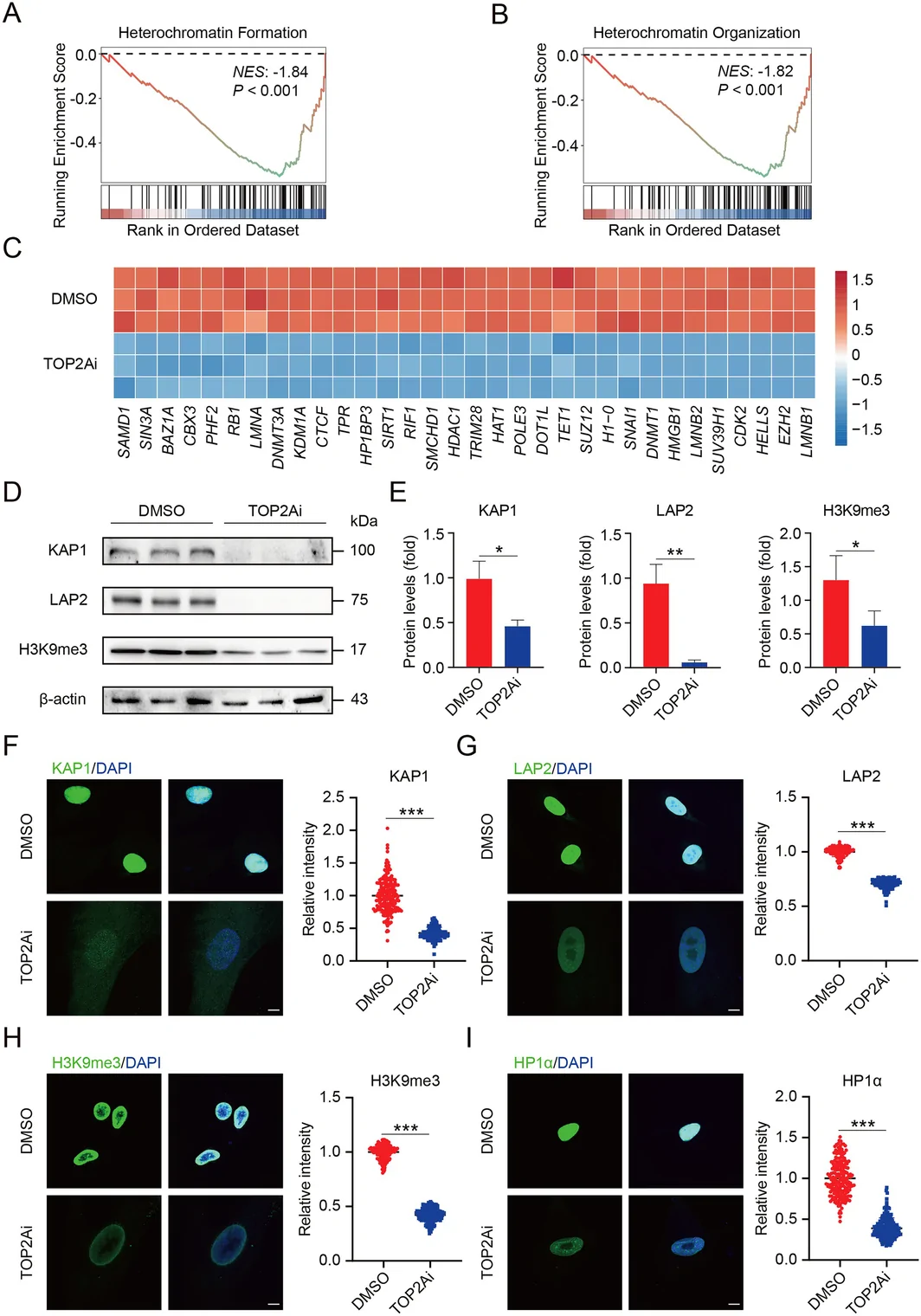
Depletion of TOP2A leads to disruption of heterochromatin integrity. (A) GSEA analysis of heterochromatin formation. (B) GSEA analysis of heterochromatin organization. (C) Heatmap analysis of heterochromatin‐associated genes. (D) Representative western blots‐showing levels of KAP1, LAP2, and the histone mark H3K9me3. (E) Quantification of western blots by ImageJ. *N* = 3 biological repeats, KAP1 (*p* = 0.012), LAP2 (*p* = 0.002), H3K9me3 (*p* = 0.042). (F–I) Immunofluorescence staining of KAP1 (F), LAP2 (G), H3K9me3 (H), and HP1α (I) with DAPI counterstaining. Scale bar: 10 μm. (F–I) *N* = 200 cells per group, *p* < 0.001. Statistical significance determined by the independent samples *t*‐test. Data are presented as the mean ± SD. *p < 0.05, **p < 0.01, ***p < 0.001. Source data are available online for this figure.

### 2.4 TOP2A Regulates *LMNB1* Expression to Control Cellular Senescence

To further understand the underlying mechanism through which TOP2A regulates MSC senescence, we conducted ATAC‐seq (assay for transposase‐accessible chromatin using sequencing), CUT&Tag (cleavage under targets and tagmentation, identifying the chromosomal DNA regions bound by the TOP2A protein), and RNA‐seq analyses. TOP2Ai resulted in 2134 up‐ and 1790 down‐regulated genes at the transcriptional level. Since TOP2A functions as a topoisomerase that modulates chromatin structure, depletion of TOP2A may cause MSC senescence by altering chromatin accessibility. Transcription Start Site (TSS) enrichment (Diao et al. 2021b) obtained from the CUT&Tag data demonstrated a higher enrichment of TOP2A at TSSs (Figure 4A). We found that the TOP2A‐targeted sequences in the CUT&Tag data were mainly located in distal intergenic regions and introns, and at a minor subset of promoters, UTRs, and exons (Figure 4B), suggesting dual roles of TOP2A in maintaining global chromatin architecture and locally facilitating transcription initiation. We intersected the results from RNA‐seq (67 genes, LogFC < −6), ATAC‐seq (15,693 genes, LogFC < 0), and CUT&Tag (2200 genes). Following the application of adjusted thresholds, we identified five core candidate genes: marker of proliferation Ki‐67 ( *MKI67* ), Lamin B1 ( *LMNB1* ), DNA meiotic recombinase 1 ( *DMC1* ), disheveled, Egl‐10, pleckstrin domain containing 1 ( *DEPDC1* ), and claspin ( *CLSPN* ) (Figure 4C). In addition, reductions in the heights of the peaks near the TSS of *LMNB1* indicate a decrease in chromatin accessibility at the TSS of *LMNB1* (Figure 4D). The other four genes also displayed decreased chromatin accessibility at TSS, which was confirmed by RNA‐seq expression data (Figure S5A,B). These results suggest that TOP2A likely targets these genes and modulates their chromatin accessibility. Except for *MKI67* , targeting of *DMC1* , *DEPDC1* , and *CSLPN* were further corroborated by CUT&Tag qPCR methodology (Figure S5C). Among these five genes, only *LMNB1* is directly involved in maintaining nuclear structural stability and chromatin organization, and its downregulation serves as both a core phenotypic hallmark and a driving factor in cellular senescence (Shah et al. 2013). Consequently, we sought to investigate whether TOP2A directly interacts with *LMNB1* to modulate MSC senescence. TOP2A directly regulated two promoter sequences and two enhancer sequences for *LMNB1*, as demonstrated through ATAC‐seq and CUT&Tag analyses. Indeed, our findings indicate that all four components of the TOP2A‐targeted *LMNB1* gene were enriched in the MSC‐TOP2A group when we conducted CUT&Tag qPCR‐amplification of these components (Figure 4E,F). This finding further confirms that TOP2A specifically targets and binds to the two promoter and enhancer sequences of *LMNB1* .

**FIGURE 4:**
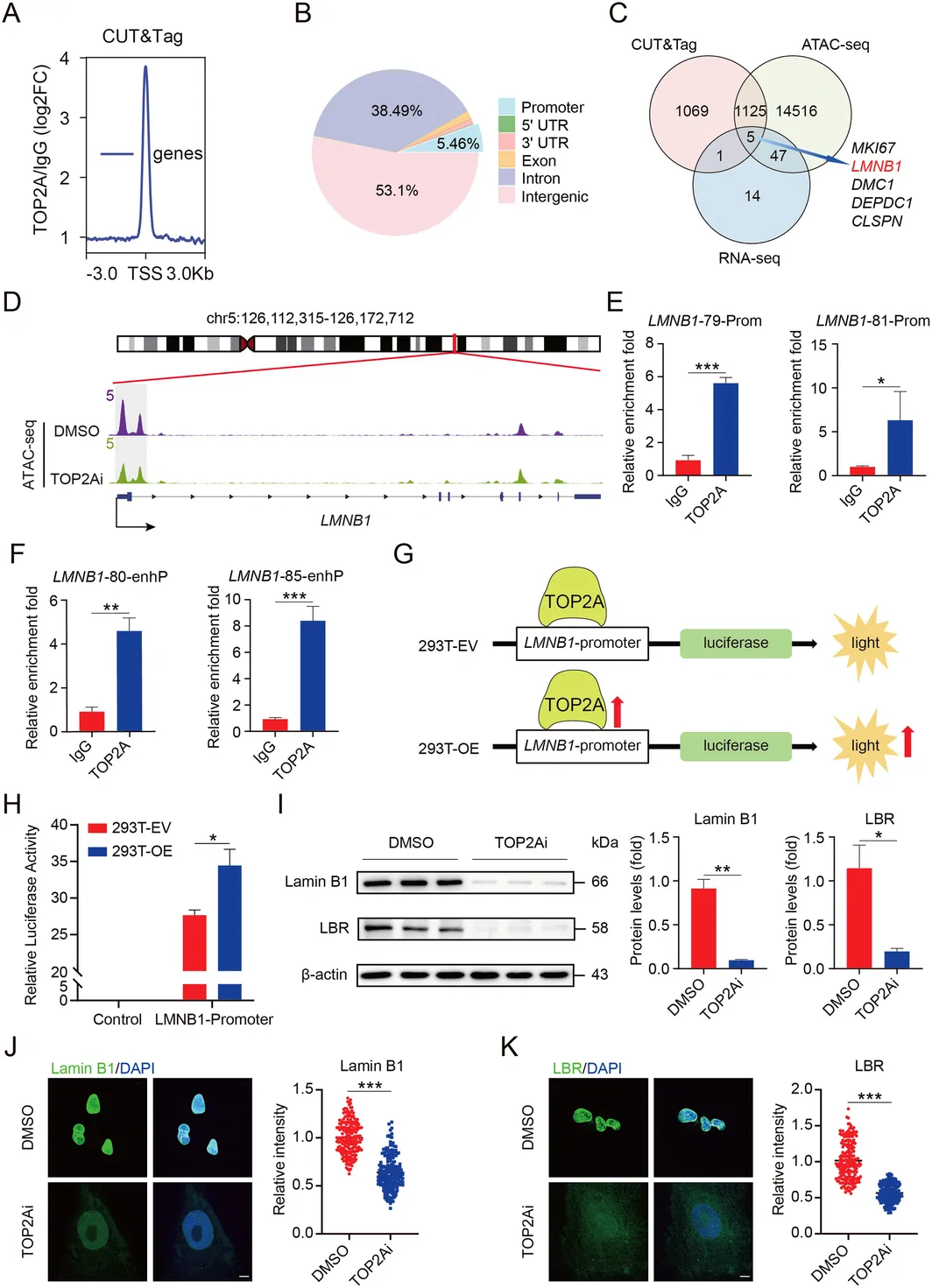
Genomic targeting of *LMNB1* by TOP2A in senescent MSCs. (A) CUT&Tag signal profiles around transcription start sites (TSSs) in MSCs. (B) Genomic distribution of TOP2A binding sites categorized by genomic region type. (C) Venn diagram showing the overlap of RNA‐seq, CUT&Tag and ATAC‐seq to identify five core candidate genes associated with chromatin alterations. (D) ATAC‐seq tracks showing chromatin accessibility changes at the *LMNB1* locus. (E) CUT&Tag‐qPCR validation of TOP2A binding to the *LMNB1* promoter region. *N* = 3 biological repeats, *LMNB1*‐79‐Prom (*p* < 0.001), *LMNB1*‐81‐Prom (*p* = 0.048). (F) CUT&Tag‐qPCR validation of TOP2A binding to an *LMNB1* enhancer region. *N* = 3 biological repeats, *LMNB1*‐80‐enhP (*p* = 0.004), *LMNB1*‐85‐enhP (*p* < 0.001). (G) Schematic of the *LMNB1* promoter luciferase reporter system. (H) Dual luciferase reporter gene assay demonstrating TOP2A‐dependent *LMNB1* promoter activity. *N* = 3 biological repeats, *p* = 0.025. (I) Western blot analysis of Lamin B1 and LBR protein levels in TOP2Ai‐treated MSCs. *N* = 3 biological repeats, Lamin B1 (*p* = 0.005), LBR (*p* = 0.023). (J) Immunofluorescence staining of Lamin B1 (green) with DAPI. Scale bar: 10 μm. *N* = 200 cells per group, *p* < 0.001. (K) Immunofluorescence staining of LBR (green) with DAPI. Scale bar: 10 μm. *N* = 200 cells per group, *p* < 0.001. Statistical significance was determined by the independent samples *t*‐test. Data are presented as the mean ± SD. *p < 0.05, **p < 0.01, ***p < 0.001. Source data are available online for this figure.

To further verify that TOP2A directly modulates the *LMNB1* promoter and regulates the expression of *LMNB1* , we conducted dual luciferase reporter gene assays. Initially, we established a FLAG‐tagged TOP2A‐overexpressing 293T cell line (293T‐OE) and an empty vector control cell line (293T‐EV). The pGL3‐basic vector and a pGL3‐LMNB1 promoter‐driven firefly luciferase plasmid were co‐transfected with a pGL3‐Renilla luciferase internal reference plasmid into 293T‐EV or 293T‐OE cells. Fluorescence intensity was measured utilizing a dual luciferase reporter gene kit. Transfection into 293T‐OE cells resulted in a stronger fluorescence signal compared to that observed in 293T‐EV cells (Figure 4G,H). Simultaneously, we found that protein expression of Lamin B1 and the Lamin B receptor (LBR) was down‐regulated in the TOP2Ai treatment group, based on western blotting and immunofluorescence staining (Figure 4I–K). Altogether, these results demonstrate that TOP2A plays a role in regulating cellular senescence through the direct modulation of *LMNB1* expression.

### 2.5 *LMNB1* Acts as a Key Downstream Effector of TOP2A in Maintaining Heterochromatin

To determine whether TOP2A specifically regulates *LMNB1*, we selected other members of the nuclear lamina family, including *LMNB2* and *LMNA/C*, as well as key heterochromatin regulators—the methyltransferase SUV39H1 and the heterochromatin “reader” and effector *CBX5* (encoding HP1α)—as indicators of heterochromatin status. We then examined the effects of TOP2A downregulation on these additional genes associated with the nuclear lamina and heterochromatin (*LMNB2*, *LMNA/C*, *SUV39H1*, and *CBX5*) (Djeghloul et al. 2016; Herrera et al. 2023; Robin and Magdinier 2016; Zhang et al. 2015), Our results demonstrated that TOP2A knockdown in MSCs significantly reduced the protein levels of Lamin B1 and heterochromatin‐associated factors (H3K9me3 and KAP1), while the expression of Lamin B2, Lamin A/C, and SUV39H1 was only mildly affected (Figure 5A,B). Combined with our findings from ATAC‐seq, CUT&Tag, and CUT&Tag‐qPCR (Figure 4D–F), as well as the dual‐luciferase reporter assay demonstrating TOP2A‐dependent *LMNB1* promoter activity (Figure 4G,H), these data confirm that TOP2A specifically targets and modulates *LMNB1*. Expression levels of HP1α (encoded by *CBX5*) were significantly downregulated following either *TOP2A* or *LMNB1* knockdown. This finding is consistent with recent reports by Hervé et al., who observed that reduced Lamin B1 levels are accompanied by decreased HP1α levels and a loss of H3K9me3‐marked heterochromatin (Herve et al. 2025). Importantly, combined knockdown of *TOP2A* and *LMNB1* (siTOP2A + siLMNB1) reduced the expression of HP1α, KAP1, H3K9me3. The magnitude of this reduction was comparable to that observed with *LMNB1* knockdown alone, suggesting that *LMNB1* is a critical node in the TOP2A‐mediated regulation of heterochromatin. To further validate whether *LMNB1* serves as a key mediator in this process, we performed rescue experiments by overexpressing *LMNB1* in TOP2A‐knockout MSCs. The results indicated that *LMNB1* overexpression restored the expression of heterochromatin‐associated factors (HP1α, KAP1, and H3K9me3) in the context of TOP2A loss (Figure 5C,D). These findings support the notion that *LMNB1* functions as a primary downstream effector of TOP2A in regulating heterochromatin stability.

**FIGURE 5:**
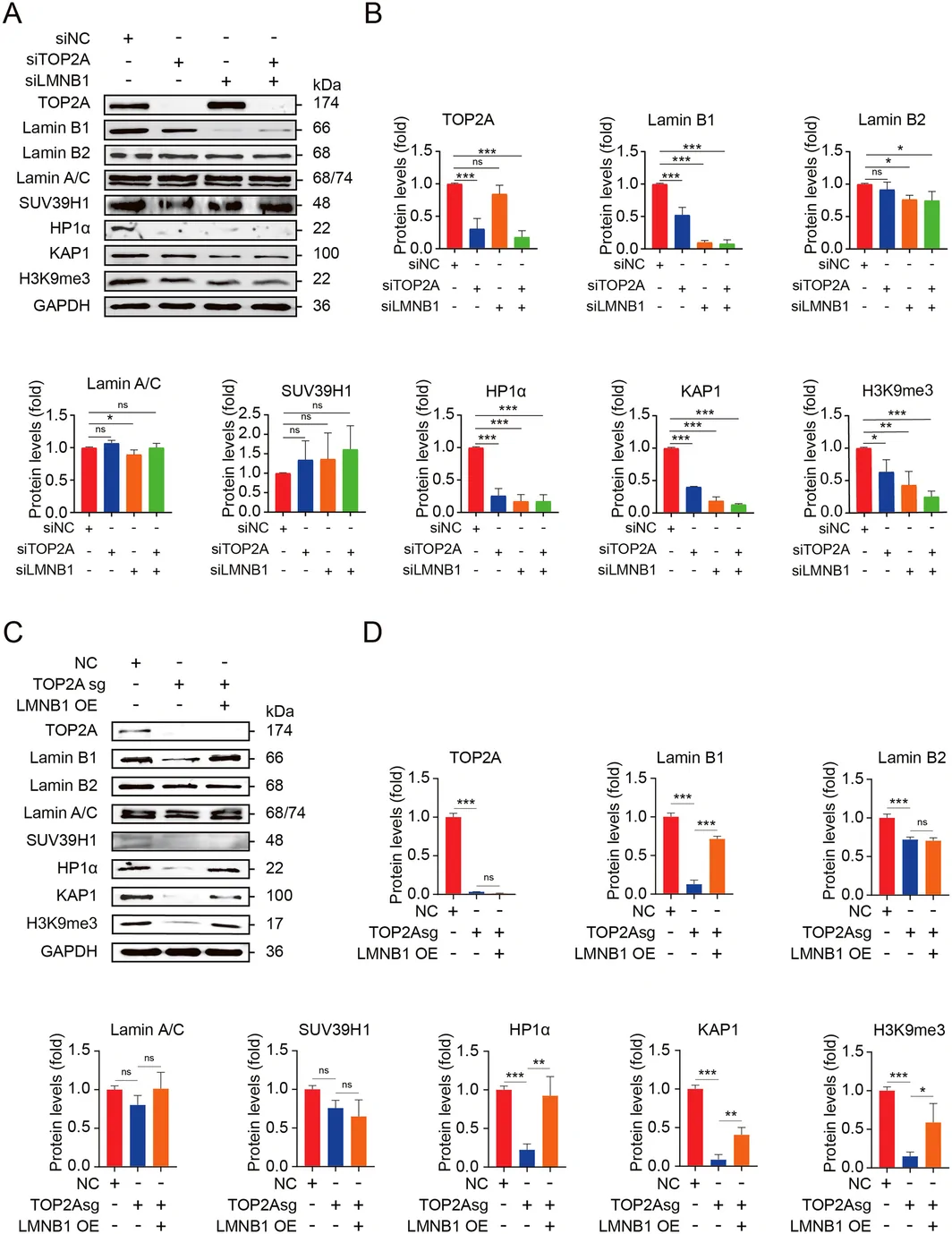
TOP2A regulates heterochromatin and nuclear lamina protein stability predominantly via *LMNB1*, as revealed by knockdown and rescue experiments. (A, B) Western blot analysis of TOP2A, nuclear membrane (Lamin B1) and heterochromatin markers and associated proteins (Lamin B2, Lamin A/C, SUV39H1, HP1α, KAP1, and H3K9me3). *N* = 3 biological repeats, TOP2A (*p* < 0.001, *p* = 0.144, *p* < 0.001), Lamin B1 (*p* < 0.001, *p* < 0.001, *p* < 0.001), Lamin B2 (*p* = 0.325, *p* = 0.018, *p* = 0.013), Lamin A/C (*p* = 0.162, *p* = 0.048, *p* = 0.977), SUV39H1 (*p* = 0.438, *p* = 0.413, *p* = 0.183), HP1α (*p* < 0.001, *p* < 0.001, *p* < 0.001), KAP1 (*p* < 0.001, *p* < 0.001, *p* < 0.001), H3K9me3 (*p* = 0.016, *p* = 0.002, *p* < 0.001). (C, D) Western blot analysis of TOP2A, nuclear membrane (Lamin B1) and heterochromatin markers and associated proteins (Lamin B2, Lamin A/C, SUV39H1, HP1α, KAP1, LAP2, and H3K9me3). *N* = 3 biological repeats, TOP2A (*p* < 0.001, *p* = 0.413.), Lamin B1 (*p* < 0.001, *p* < 0.001), Lamin B2 (*p* < 0.001, *p* = 0.678), Lamin A/C (*p* = 0.143, *p* = 0.124), SUV39H1 (*p* = 0.078, *p* = 0.379), HP1α (*p* < 0.001, *p* = 0.001), KAP1 (*p* < 0.001, *p* = 0.002), H3K9me3 (*p* < 0.001, *p* = 0.011). Statistical significance was determined by the One‐way ANOVA. Data are presented as the mean ± SD. *p < 0.05, **p < 0.01, ***p < 0.001. Source data are available online for this figure.

### 2.6 Overexpression of TOP2A Counteracts Cellular Senescence

To further investigate the functional role of TOP2A in MSC senescence, we assessed the potential of TOP2A overexpression to mitigate senescence‐associated phenotypes in MSCs. We constructed a TOP2A‐overexpressing cell line denoted MSC‐OE, and empty vector control cell line MSC‐EV. The MSC‐OE and MSC‐EV cells were subjected to serial replicative passaging. As passaging continued, we observed that MSC‐EV cells exhibited a higher degree of senescence compared to MSC‐OE cells, as evidenced by increased granularity, larger and flatter morphology, and higher SA‐β‐gal positivity (Figure 6A), suggesting that overexpression of TOP2A can reduce the rate of MSC senescence. Furthermore, we assessed the colony forming ability of MSC‐OE and MSC‐EV cells by crystal violet staining. As expected, the extent of colony formation of MSC‐OE cells was superior to that of MSC‐EV cells (Figure 6B). We further compared the expression of nuclear membrane and heterochromatin‐associated proteins between MSC‐OE and MSC‐EV cells by western blotting. Expression of nuclear membrane‐associated proteins Lamin B1 and LBR was up‐regulated in MSC‐OE cells, as were the heterochromatin markers and associated proteins, H3K9me3, LAP2, and KAP1 (Figure 6C,D). Furthermore, immunofluorescence staining for Lamin B1, LBR, H3K9me3, and LAP2 displayed the same trend as the western blotting results for MSC‐OE and MSC‐EV cells (Figure 6E–I). Taken together, these results indicate that overexpression of TOP2A slowed down the loss of nuclear membrane and heterochromatin‐associated proteins, and thus retarded the progression of MSC senescence by promoting LAD maintenance and stabilizing heterochromatin, which has been verified to play a crucial role in the counteracting senescence of MSCs (Bi et al. 2020; Diao et al. 2021a; Liang et al. 2021; Zhao et al. 2022). Furthermore, the size of senescent cell nuclei is usually enlarged as a result of the loss of nuclear membrane and heterochromatin proteins (Efremov et al. 2022; Schibler et al. 2023). Overexpression of TOP2A sustained the morphological integrity of the nucleus and mitigated nuclear enlargement. The average nuclear size of MSC‐OE was smaller than that of MSC‐EV cells (*p* < 0.01) (Figure 6J).

**FIGURE 6:**
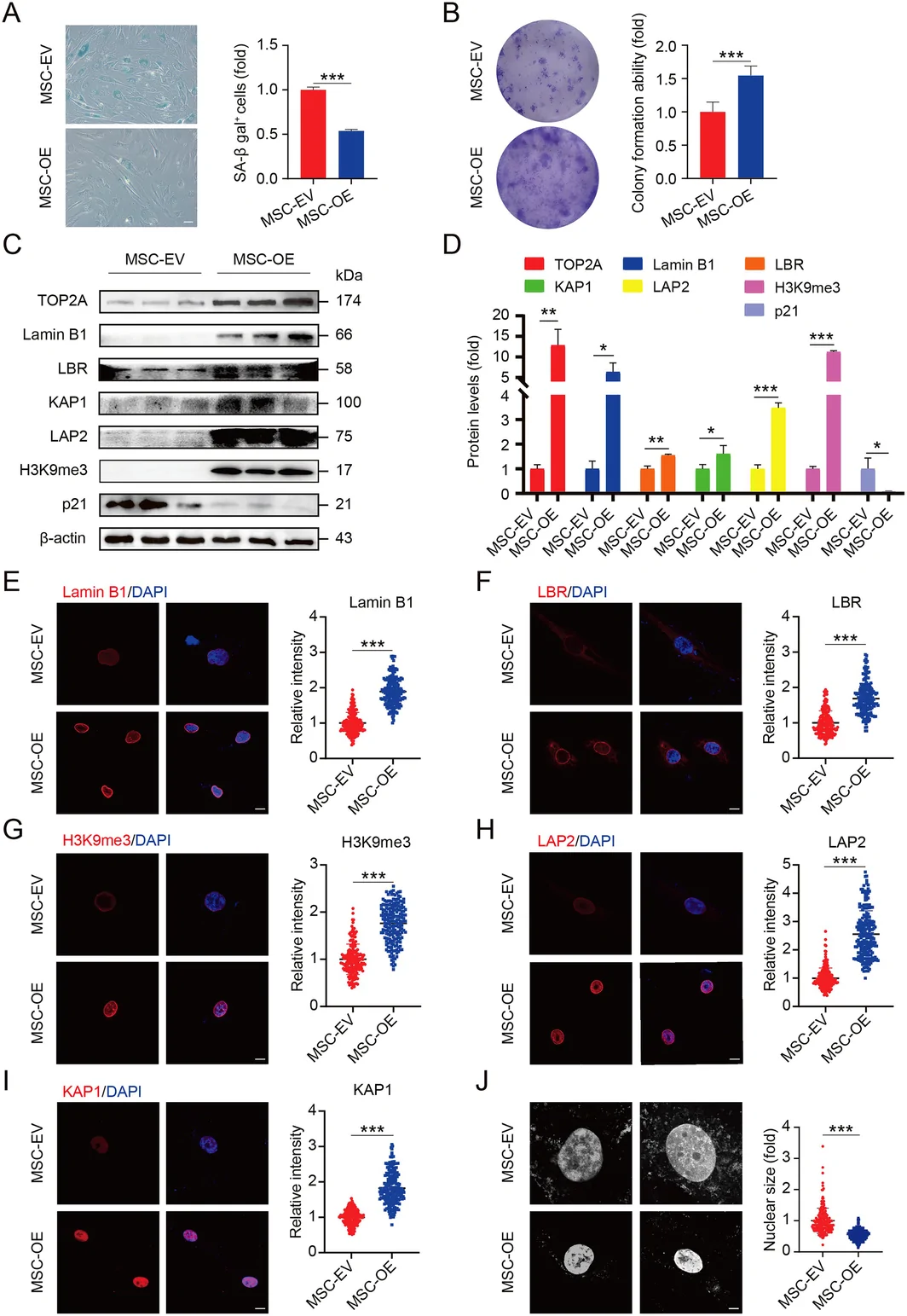
Functional rescue of the senescence phenotypes by TOP2A overexpression. (A) SA‐β‐gal staining comparing empty vector control (MSC‐EV) and TOP2A‐overexpressing MSCs (MSC‐OE). Scale bar: 50 μm. *N* = 5 biological repeats, *p* < 0.001. (B) Colony formation assay of MSC‐EV and MSC‐OE cells. *N* = 3 biological repeats, *p* < 0.001. (C) Western blot analysis of nuclear membrane (Lamin B1, LBR) and heterochromatin markers and associated proteins (KAP1, LAP2, and H3K9me3). (D) Densitometric quantification of protein levels. *N* = 3 biological repeats, TOP2A (*p* = 0.006), Lamin B1 (*p* = 0.013), LBR (*p* = 0.002), KAP1 (*p* = 0.023), LAP2 (*p* < 0.001), H3K9me3 (*p* < 0.001), p21 (*p* = 0.022). (E) Immunofluorescence staining of Lamin B1 (red) with DAPI. Scale bar: 10 μm. *N* = 200 cells per group, *p* < 0.001. (F) Immunofluorescence staining of LBR (red) with DAPI. Scale bar: 10 μm. *N* = 200 cells per group, *p* < 0.001. (G) Immunofluorescence staining of H3K9me3 (red) with DAPI. Scale bar: 10 μm. *N* = 200 cells per group, *p* < 0.001. (H) Immunofluorescence staining of LAP2 (red) with DAPI. Scale bar: 10 μm. *N* = 200 cells per group, *p* < 0.001. (I) Immunofluorescence staining of KAP1 (red) with DAPI. Scale bar: 10 μm. *N* = 200 cells per group, *p* < 0.001. (J) Nuclear area quantification of MSC‐EV and MSC‐OE cells. Scale bar: 5 μm. *N* = 200 cells per group, *p* < 0.001. Statistical significance was determined by the independent samples *t*‐test. Data are presented as the mean ± SD. *p < 0.05, **p < 0.01, ***p < 0.001. Source data are available online for this figure.

Maintenance of mitochondrial membrane potential is critical for the normal function of mitochondria and mitochondrial dysfunction is one of the hallmarks of aging (Lopez‐Otin et al. 2023; Miwa et al. 2022). We subjected the same generation of MSC‐OE and MSC‐EV cells to mitochondrial membrane potential staining. The fluorescence intensity of Mito‐Tracker Red CMXRos in MSC‐OE cells was greater than that in MSC‐EV cells, indicating that the mitochondrial membrane potential of MSC‐OE cells was higher than that in MSC‐EV cells (Figure S6A). This suggests that the mitochondrial function of MSC‐OE cells was preserved by TOP2A. In addition, EdU staining was conducted on identically passaged MSC‐OE and MSC‐EV cells, revealing a significantly higher number of EdU‐positive cells in MSC‐OE cells (Figure S6B), reflecting greater cellular proliferative activity in MSC‐OE cells. Overall, overexpression of TOP2A promotes LAD maintenance and mitigates the loss of nuclear membrane and heterochromatin proteins in MSCs, thereby retarding cellular senescence and preserving both mitochondrial membrane potential and proliferative capacity of the cells.

### 2.7 TOP2Ai Induces Aging of C57BL/6J Mice

As established above, we confirmed that TOP2A sustains *LMNB1* transcription to stabilize heterochromatin and counteract senescence. Given that Lamin B1 plays a critical role in aging‐related neurodegenerative diseases (Bedrosian et al. 2021; Bin Imtiaz et al. 2021), and considering our analysis of public databases showing age‐associated downregulation of TOP2A not only in peripheral tissues (e.g., spleen, liver) but also critically in the brain (Figure S3C), these collective findings prompted us to investigate whether targeted depletion of TOP2A in vivo could similarly induce senescence in C57BL/6J mice, with particular interest in its potential impact on brain aging.

Therefore, we established two mouse models, involving natural aging (6‐ to 8‐week mice as young mice, 20‐month‐old mice as old mice) and TOP2Ai‐induced aged (10‐week‐old mice, TOP2Ai‐treated for two months) C57BL/6J mice (Figure 7A), and conducted behavioral experiments (Figure 7B). Hair thinning and loss are prominent characteristics of aging (Lei and Chuong 2016; Li et al. 2020). Natural aging of C57BL/6J mice is accompanied by significant hair density reduction, increased hair shaft roughness and diminished light reflectance (Figure 7C). TOP2Ai‐induced aging of C57BL/6J mice resulted in a phenotype similar to natural aging of C57BL/6J mice (Figure 7D). Previous research has indicated an involvement of TOP2A in neurogenesis (Ledvin et al. 2023; Qin et al. 2022; Roy et al. 2022). SA‐β‐gal‐positive cells in the hippocampal region increases with age (Zhang, Li, et al. 2021) We indeed found that the hippocampal region and cortical layers of the brains of old mice exhibited a higher number of SA‐β‐gal‐positive cells with distinct perinuclear blue staining, corroborating previous reports (Figure 7E,F and Figure S7A,B). A similar trend was observed in the hippocampal region and cortical layers of mouse brain tissues from mice treated with TOP2Ai for 2 months (Figure 7G,H and Figure S7C,D). These results imply that downregulation of TOP2A might accelerate aging in the hippocampi and cortical layers of the mouse brain.

**FIGURE 7:**
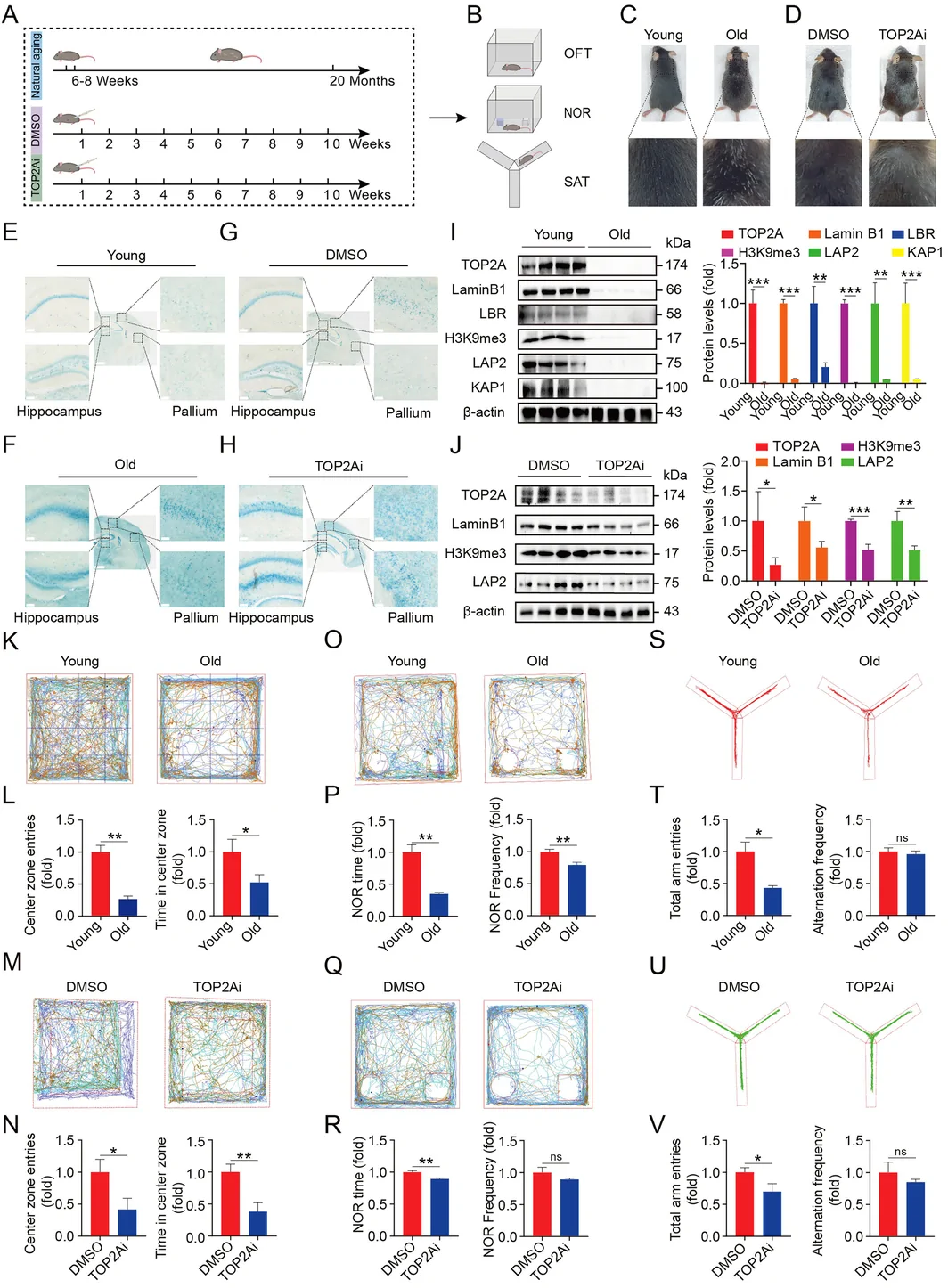
Systemic senescence phenotypes in TOP2Ai‐induced aging mice. (A) Schematic representation of the construction of both the natural aging and the TOP2Ai‐induced aging mouse models. (B) Behavioral assessments, including the open field test (locomotor activity) and new object recognition test (memory). (C) Representative images of age‐related alopecia progression of natural aging mice. (D) Representative images of age‐related alopecia progression of TOP2Ai‐treated mice. (E, F) SA‐β‐gal staining in the hippocampal regions and cortical layers of the brains of naturally aging mice. (G, H) SA‐β‐gal staining in the hippocampal regions and cortical layers of brains of TOP2Ai‐treated mice. (E–H) scale bar: 800 μm (half brain), scale bar: 100 μm (enlarged brain section). (I) Western blot analysis of Lamin B1, LBR, H3K9me3, LAP2 and KAP1 protein levels in brain tissue of natural aging mice. *N* = 4 biological repeats, TOP2A (*p* < 0.001), Lamin B1 (*p* < 0.001), LBR (*p* = 0.004), H3K9me3 (*p* < 0.001), LAP2 (*p* = 0.005), KAP1 (*p* < 0.001). (J) Western blot analysis of the same protein panel for TOP2Ai‐treated mice. *N* = 4 biological repeats, TOP2A (*p* = 0.027), Lamin B1 (*p* = 0.013), H3K9me3 (*p* < 0.001), and LAP2 (*p* = 0.004). (K, L) Open field test quantification: Locomotor trajectories (K), and entries and time in the center zone (L) for natural aging mice. *N* = 3 biological repeats, (*p* = 0.002, *p* = 0.031). (M, N) Open field test of TOP2Ai‐treated mice: Trajectories (M), entries and time in the center zone (N). *N* = 3 biological repeats, (*p* = 0.020, *p* = 0.005). (O, P) Novel object recognition test: Locomotor trajectories (O), recognition index (P) by natural aging mice. (*p* = 0.003, *p* = 0.009). (Q, R) Novel object recognition by TOP2Ai‐treated mice: Locomotor trajectories (Q), recognition index (R). (*p* = 0.007, *p* = 0.097). (S, T) Y‐maze test (spatial memory) in natural aging mice. Shown are the number of alternations and the frequency of alternations in the ABC arm. (*p* = 0.017, *p* = 0.404). (U, V) Y‐maze test (spatial memory) in TOP2Ai‐treated mice. Shown are the number of alternations and frequency of alternations in the ABC arm. (*p* = 0.033, *p* = 0.254). Statistical significance was determined by the independent samples *t*‐test. Data are presented as the mean ± SD. *p < 0.05, **p < 0.01, ***p < 0.001. Source data are available online for this figure.

Furthermore, we examined whether depletion of TOP2A contributes to senescence by decreasing nuclear membrane and heterochromatin‐associated proteins in brain tissues of mice. As expected, a decrease in the expression level of nuclear membrane‐associated proteins Lamin B1 and LBR, and heterochromatin markers and associated proteins, including H3K9me3, LAP2, and KAP1, was all down‐regulated alongside the reduction of TOP2A in the brain tissues of naturally aged mice (Figure 7I). Similarly, in the brains of TOP2Ai‐treated mice, the expression level of TOP2A, Lamin B1, H3K9me3, and LAP2 was all down‐regulated (Figure 7J). This evidence further supports that depletion of TOP2A in mice might contribute to brain aging through the reduction of nuclear membrane and heterochromatin‐associated proteins. Furthermore, in TOP2Ai‐induced aging mice, we observed signs of aging in multiple organs such as mesoderm‐derived tissues (heart, adipose, kidney), ectoderm‐derived tissues (skin), accompanied by decreased levels of TOP2A, Lamin B1, and nuclear membrane/heterochromatin‐associated markers (KAP1, H3K9me3). (Figure S8A–D).

We further evaluated aging phenotypes in old (20‐month‐old) and TOP2Ai‐treated mice. Open field tests revealed that old mice exhibited reduced movement trajectories, shorter travel distances, peripheral preference, and diminished exploratory behavior compared to 6‐ to 8‐week‐old mice (Figure 7K,L). TOP2Ai‐treated mice displayed similar declines in locomotor activity (Figure 7M,N). Novel object recognition tests showed reduced exploration time/frequency for new objects in both natural aging and TOP2Ai groups (Figure 7O–R). Y‐maze tests demonstrated decreased spontaneous alternation behaviors in natural aging and TOP2Ai‐treated mice, indicating impaired spatial memory (Figure 7S–V). Collectively, TOP2A inhibition recapitulated key aging‐associated behavioral deficits, including motor decline, reduced exploration, and spatial memory impairment, suggesting accelerated aging in TOP2Ai‐treated C57BL/6J mice.

## 3 Discussion

Adult stem cell exhaustion, marked by functional decline and regenerative failure, is a central driver of organismal aging (Brunet et al. 2023; Navarro Negredo et al. 2020). Our study unveils TOP2A as a novel functional regulator of MSC senescence, operating through sustaining Lamin B1 expression to preserve nuclear architecture and heterochromatin stability. We demonstrate that TOP2A downregulation reduces the expression of Lamin B1, leading to nuclear envelope collapse and loss of heterochromatin markers (e.g., H3K9me3, KAP1, LAP2), followed by a state of heterochromatin instability, which collectively drive senescence‐associated phenotypes (e.g., p21/p16 accumulation, SASP). Furthermore, depletion of TOP2A led to a decrease in mitochondrial membrane potential, cell proliferation, colony formation and trilineage differentiation ability of MSCs, which highlight the critical role of TOP2A in sustaining functional homeostasis of MSCs. Conversely, *TOP2A* overexpression rescues Lamin B1 levels and nuclear integrity, mitigating senescence. Strikingly, TOP2A inhibition in mice recapitulates brain aging and neurodegenerative‐like behavioral phenotypes, mirroring heterochromatin erosion and SA‐β‐gal activation observed in naturally aging brains. These findings establish TOP2A as a key regulator of MSC senescence, and further suggest that TOP2A contributes to brain aging in vivo, potentially through mechanisms involving nuclear envelope integrity and heterochromatin stability.

TOP2A is a DNA topoisomerase, primarily involved in unwinding the supercoiled structure of double‐stranded DNA during DNA replication and transcription by cleaving and rejoining the DNA double strands. Notably, TOP2A plays a critical role in establishing mitotic chromosome cohesion (Meijering et al. 2022; Nielsen et al. 2020) and maintaining genomic stability (Tian et al. 2021; Zhang et al. 2020). Previous understanding of TOP2A was mainly in the fields of cell cycle regulation and tumorigenesis but recently researchers have found that it plays a role in proliferation, migration, invasion, and development of both embryonic stem cells and adult neural stem cells (Dalcher et al. 2020; Duan et al. 2022; Hu et al. 2020; Qin et al. 2022). Survival of human pluripotent stem cells can be modulated by application of small molecules that target TOP2A (Ben‐David et al. 2015). Furthermore, short‐term expression of factors, such as OCT‐3/4, SOX2, KLF4, and c‐Myc, has been shown to enhance liver regeneration through TOP2A‐mediated partial reprogramming (Hishida et al. 2022). TOP2A deficiency in human endometrial stem cells affected the processes of decidualization and embryo implantation, leading to recurrent implantation failure (Fu et al. 2022). However, the role of TOP2A in regulating stem cell senescence remains elusive.

Our study shows TOP2A regulates nuclear architecture by directly binding to the promoter region of *LMNB1* to regulate *LMNB1* expression, contributing to nuclear lamina stability, thereby regulating its expression. As a nuclear envelope protein, *LMNB1* is not only an essential scaffold for heterochromatin organization, but also participates in the regulation of gene expression through interactions with the genome via lamina‐associated domains (LADs) (Wong et al. 2022). When the level of Lamin B1 is reduced, it compromises the cell's DNA repair capacity, leading to chromatin instability (Butin‐Israeli et al. 2015; Liu et al. 2015). The loss of Lamin B1 is associated with the disruption of perinuclear heterochromatin organization, accompanied by the spatial redistribution and altered levels of heterochromatin markers, such as H3K9me3 (a repressive histone mark) and H3K27me3 (Camps et al. 2014; Sadaie et al. 2013; Shah et al. 2013). Previous studies have already demonstrated that the instability of heterochromatin is an important characteristic of cellular senescence, and the reduction of *LMNB1* is associated with aging (Bedrosian et al. 2021; Bin Imtiaz et al. 2021; Lammerhirt et al. 2022; Matias et al. 2022).

Our results indicate that downregulation of TOP2A leads to the downregulation of *LMNB1*, which in turn results in downregulation of the heterochromatin marker H3K9me3 and the heterochromatin stabilizers KAP1 and LAP2 in MSCs. Likewise, in the hippocampus and cortical layers of mice aged naturally or by TOP2Ai treatment, Lamin B1, H3K9me3, KAP1, and LAP2 are also downregulated concomitant with an increase in SA‐β‐gal‐positive cells. These correlative observations raise the possibility that the TOP2A–*LMNB1* axis operates in the brain during aging. However, it remains to be determined whether TOP2A regulates brain aging primarily through this axis or via other TOP2A‐dependent molecular mechanisms (e.g., direct regulation of heterochromatin‐related genes, DNA topoisomerase activity‐mediated genomic stability, or distinct downstream effectors in neurons versus MSCs). Dissecting these possibilities will require cell‐type‐specific manipulation of TOP2A and *LMNB1* in the brain.

Therefore, regulation of TOP2A on Lamin B1 may ensure the stability and highly condensed state of heterochromatic regions, thereby suppressing the activation of aging‐related genes. In addition, TOP2A may regulate the dynamic balance of the nuclear scaffold through *LMNB1* , maintaining the delicate connection between the nuclear envelope and heterochromatin. Therefore, our findings indicate that in MSCs, TOP2A functions as an epigenetic stabilizer, and that targeting its depletion may disrupt nuclear structural integrity and heterochromatin homeostasis.

This study, for the first time, reveals the critical intermediary role of TOP2A in MSC senescence. This finding supplements our understanding of the synergistic role of TOP2A and nuclear envelope proteins in maintaining genomic stability. Although we have identified a role for TOP2A in maintaining heterochromatin stability in MSCs, further research is needed to elucidate its molecular mechanisms. For example, how does TOP2A precisely maintain heterochromatin balance and what are the upstream regulatory genes of TOP2A? Furthermore, whether this regulatory mechanism is conserved in other cell types or tissues, such as neurons or hepatocytes, warrants dedicated investigation. These questions remain to be answered.

Taken together, this study positions TOP2A as a linchpin connecting DNA topology, nuclear architecture, and MSC senescence. By orchestrating *LMNB1*‐mediated heterochromatin stability, TOP2A safeguards against senescence in MSCs. This finding not only deepens our understanding of the synergistic role of TOP2A and nuclear envelope proteins in maintaining genomic stability, but also illuminates a promising avenue for aging interventions, specifically, targeting nuclear scaffold resilience to restore stem cell vitality.

## 4 Materials and Methods

### 4.1 Ethics Approval and Consent to Participate

All animal experiments and procedures were approved by the Animal Ethics Committee of Shantou University Medical College Animal Center (SUMCSY2024‐014). The use of human umbilical cord for isolating MSCs was approved by the Ethics Committee of Shantou University Medical College (SUMC2021‐89).

### 4.2 Isolation and Cultivation of Umbilical Cord Mesenchymal Stem Cells

Ethical approval for this study was obtained from the Ethics Committee of Shantou University Medical College. Human umbilical cord samples were donated by three consenting donors at the Second Affiliated Hospital of Shantou University School of Medicine. These donors delivered full‐term infants via cesarean section. The umbilical cords were subjected to three washes with PBS, followed by disinfection in iodine solution for 30 s, and subsequently washed three times with sterile PBS. The specimens were then cut into small sections measuring 2–3 cm, and any bruised areas were removed using flat forceps. The arteries and veins were excised, then the Wharton's jelly was separated using toothed forceps and cut into 1 mm^3^ pieces with ophthalmic scissors. A 0.05% solution of collagenase IV (Gibco, 17104019) was utilized for digestion in an incubator at 37°C for 90 min. Following digestion, the supernatant was removed via centrifugation, and 0.05% trypsin (Thermo Fisher, 25200072) was added for an additional 15 min of digestion at 37°C in a water bath. The cells were then washed twice with DMEM, resuspended in complete medium, and distributed into 6‐well plates for incubation. Medium was changed after 3 days and subsequently every 2 days. When cell growth reached 80%–90% confluence, the cells were passaged at a ratio of 1:6 and cryopreserved in a solution of DMEM: FBS: DMSO (6:3:1).

### 4.3 Animals

All animal experiments and procedures were approved by the Ethics Committee of the Animal Center of Shantou University Medical College. Male C57BL/6J mice (6–8 weeks old) were purchased from the Beijing Vital River Laboratory Animal Technology Co. Mice were maintained in an environment free from specific pathogens. A natural aging model was established using mice at 20 months of age. All mice were provided with a standard diet and were housed under a 12/12‐h light/dark cycle. Prior to experimentation, mice underwent an acclimatization period of 1–2 weeks. Each cage housed four mice, with bedding changed and feed and water replenished weekly. Health status of all mice was regularly monitored, and no behavioral phenotypes were observed.

### 4.4 C57BL/6J Aging Mouse Model

A C57BL/6J mouse model was utilized for the study of natural aging. Male mice 6–8 weeks of age were selected as young subjects, while those raised to 20 months were classified as aged subjects. Additionally, a TOP2Ai‐induced aging model was employed, wherein male C57BL/6J mice over 10 weeks of age were selected. These mice were randomized into two groups: one receiving DMSO and the other receiving TOP2Ai. Treatment involved intraperitoneal injections at a dosage of 5 mg/kg, administered twice weekly for 2 months. All procedures adhered to animal welfare regulations, and efforts were made to protect the drugs from light exposure. Injections were conducted at consistent times and in a standardized sequence. Following each injection, the mice were provided with food and water, and their bedding was maintained in a clean condition. Periodic observations of the mice were conducted throughout the aging process.

### 4.5 Analysis of Differentiation Genes Between Young and Senescent MSCs

We merged the GSE35957 and GSE30391 datasets, obtaining a combined dataset that included 22 samples, comprising 11 young and 11 senescent MSCs. After removing batch effects, we performed differential expression analysis on the merged dataset using the limma package and presented the results in the form of heatmaps and volcano plots. Using a Log2FC absolute value > 3 and an adjusted *p* < 0.05 as criteria, we identified senescence‐related candidate gene set A.

### 4.6 WGCNA Analysis

Genes with an absolute log2‐fold change (log_2_FC) greater than 0.6 from the differential analysis were used as input data, with a total of 5647 genes included in the WGCNA analysis. We selected 4 as the soft threshold to ensure the robustness of the network, and set the minimum number of genes in a module to 30 to construct the co‐expression network, resulting in a total of 18 modules. Next, we used correlation parameters to measure the association between modules and phenotypes, and visualized the results with a correlation heatmap (Figure S1E). The results showed that the MEblue and MEturquoise modules had the highest correlation coefficients, −0.93 and 0.93, respectively, and *p*‐values for both indicated statistical significance. We selected 83 genes, with absolute values of gene significance (GS) and module membership (MM) greater than or equal to 0.9, from the MEblue and MEturquoise modules as senescence‐related candidate gene set B.

### 4.7 scRNA‐Seq Data Analysis

MSC single‐cell data were from https://data.mendeley.com/datasets/f4b2ykfv56/1. The unique molecular identifier (UMI) matrix was subsequently analyzed using Seurat (version 4.3.0). We performed initial data quality control, filtering, and normalization, and then selected the top 2000 highly variable genes for dimensionality reduction and clustering analysis. With a resolution of 0.6 and the first 20 principal components (PCs) chosen for optimal clustering, we visualized the results using a UMAP plot, ultimately obtaining 8 clusters. We used MSC‐positive surface markers, namely THY1 (CD90), ENG (CD105), and NT5E (CD73), and negative surface markers CD14, CD34, PTPRC (CD45), and HLA‐DR as criteria to distinguish MSC clusters from non‐MSC clusters. The results showed that the quality of the single‐cell data was good. Differential gene expression analysis was conducted across clusters generated at various resolutions through the Wilcoxon rank sum test, using the FindMarkers function. We used multiple indicators, including proliferation markers *MKI67* and *PCNA* , senescence marker *CDKN1A* , and senescence‐associated secretory phenotype (SASP) genes *SERPINE2* and *MMP2* , to identify young or senescent cell subpopulations. Compared with other clusters, clusters 1–3 had higher expression levels and proportions of senescence markers *CDKN1A* , *SERPINE2* , and *MMP2* . Therefore, we defined clusters 1–3 as *CDKN1A* ^+^ *MKI67* ^−^ MSCs. In contrast, clusters 4–8 highly expressed the *MKI67* , so we defined clusters 4–8 as *CDKN1A* ^−^ *MKI67* ^+^ MSCs (Extended Data Figure 1a,b). After differential analysis of the two groups, genes with *p*_val_adj < 0.05 and an absolute value of avg_log_2_FC > 1.5 were selected as senescence‐related candidate gene set C.

### 4.8 Detection of MSC Surface Markers by Flow Cytometry

We selected the following biomarkers for umbilical cord MSCs: CD105 (BioLegend, 800,503), CD105 (Beijing 4A Biotech Co, FHF105), CD90 (Beijing 4A Biotech Co, FHF090), CD73 (Beijing 4A Biotech Co, FHF073), CD45 (BioLegend, 304,006), CD34 (Beijing 4A Biotech Co, FHF034), CD14 (Beijing 4A Biotech Co, FHF014), and HLA‐DR (Beijing 4A Biotech Co, FHF HLA‐DR). Cells were fixed with 4% paraformaldehyde (Beyotime, P0099) for 15 min. Following fixation, antibodies were incubated on ice for 30 min. After the incubation period, the cells were washed with PBS. Testing was conducted using a BDC6 instrument, and the results were analyzed with FlowJo software.

### 4.9 SA‐β‐Gal Staining

We utilized a Cellular Senescence β‐Galactosidase Staining Kit (Beyotime, C0602) for the staining procedure. Specifically, cells were cultured in 12‐well plates, after which the cell culture medium was removed. The cells were washed three times with PBS, and β‐galactosidase staining fixative was added, and the cells were allowed to fix for 15 min at room temperature. Following fixation, the cells were washed three additional times with PBS. The SA‐β‐gal dye was then added to the plates, followed by placing the 12‐well plates in a light box and incubating overnight in a CO_2_ ‐free incubator at 37°C. Staining was considered complete when several blue cells were observed. Then. Staining solution was removed, and cells were washed three times with PBS. In cases where crystals formed, 70% ethanol was added, and the samples were agitated on a horizontal shaker at 140 rpm for 15 min until the crystals dissolved. After removing the 70% ethanol, PBS was added for photography or for storage at 4°C.

### 4.10 Cell Proliferation Assay

We utilized the BeyoClick EdU‐594 Cell Proliferation Assay Kit (Beyotime, C0078S) for our experiments. Cells were incubated at 37°C for 2 h with EdU at a final concentration of 10 μM, and the samples were incubated for 2 h in an incubator. Following incubation, cells were washed with PBS and subsequently fixed with 4% paraformaldehyde for 15 min at room temperature. Cell permeabilization was achieved using a 0.3% Triton X‐100 solution in PBS. Click Additive solution was prepared and used for staining, followed by DAPI staining to visualize the nuclei. Imaging was conducted using a ZEISS Observer A1 microscope (ZEISS, Axio Observer A1).

### 4.11 Colony Formation Assay

A six‐well plate was inoculated with 800 cells per well, and the culture medium was changed every 3 days. After a period of 2 weeks, the formation of colonies was observed. Colonies were subsequently fixed in 4% paraformaldehyde for 15 min at room temperature. Following fixation, cells were stained with a crystal violet solution (Beyotime, C0121) for an additional 15 min. The wells were then thoroughly washed with PBS and air‐dried in preparation for photography. Colony formation was quantified using ImageJ.

### 4.12 Q‐PCR and Western Blotting

We utilized RNAiso Plus (TaKaRa, 9109) for the extraction of RNA. Extracted RNA was subsequently reverse transcribed into cDNA using reverse transcriptase (TOYO, FSQ‐201). We used an ABI QS5 Real Time PCR (ABI, QuantStudio 5) and SYBR mix (ABI, A25742) for q‐PCR. We used RIPA buffer (BIOTRONIK, P0013B) for cell lysis and added RMSF (BIOTRONIK, ST2573) to inhibit protein degradation. We also used the BCA Protein Concentration Assay Kit (Beyotime, P0010) method to measure the protein concentration, and SDS (Beyotime, P0286) was added to denature the protein. We used a rapid gel preparation kit (Beyotime, P0012AC) for western blotting gel preparation, and NC membranes (Millipore, HATF00010) were employed for membrane transfer. We also used 5% BSA (Beyotime, ST023) for blocking. Primary antibodies were incubated overnight at 4°C, and were against TOP2A (Abcam, ab52934, 1: 1000), TOP2A (Proteintech, 66541, 1: 200), TOP2A (Proteintech, 20233–1‐AP, 1: 200), p21 (Proteintech, 10355, 1: 1000), p21 (CST, 2947T, 1: 1000), p16 (Proteintech, 10883, 1: 1000), Lamin B1 (Proteintech, 12987, 1: 1000), H3K9me3 (Abcam, ab8898, 1: 1000), LBR (Proteintech, 12398–1‐AP, 1: 1000), KAP1 (Proteintech, 66630–1‐Ig, 1: 1000), LAP2 (Proteintech, 14651–1‐AP, 1: 1000), H3K9me3 (Abcam, ab8898, 1: 1000), Lamin B2 (ABclonal, A5001, 1:1000), Lamin A/C (ABclonal, A5001, 1:1000), SUV39H1 (Proteintech, 10574–1‐A, 1:1000), GAPDH (ABclonal, A3741, 1:1000), and β‐actin (CST, 4970S, 1: 1000). Membranes were incubated with secondary antibodies at room temperature for 2 h, specifically HRP‐conjugated AffiniPure goat anti‐mouse IgG (H + L) (BOSTER, BA1050) and HRP‐conjugated AffiniPure goat anti‐rabbit IgG (H + L) (BOSTER, BA1054). Chemiluminescent detection was performed using a chemiluminescence solution (Thermo Fisher, 34076), and imaging was conducted with a Bio‐Rad ChemiDoc XRS+ chemiluminescent imaging system (Bio‐Rad, Bio‐Rad ChemiDoc XRS+). The resulting images were analyzed using Image Lab software.

### 4.13 Laser Scanning Confocal Microscopy

We inoculated the cells on coverslips (NEST, 801008) and subsequently fixed them in 4% paraformaldehyde (Beyotime, P0099). Cell permeabilization was conducted using 0.1% Triton X‐100 (Sangon, 9002) in conjunction with a 5% BSA‐PBS blocking solution. Primary antibody incubation was performed overnight at 4°C with antibodies against the following: TOP2A (Abcam, ab52934, 1:200), TOP2A (Proteintech, 66,541, 1:100), (Proteintech, 20,233–1‐AP, 1:200), p21 (Proteintech, 10,355, 1:200), p16 (Proteintech, 10,883, 1:200), Lamin B1 (Proteintech, 12,987, 1:200), H3K9me3 (Abcam, ab8898, 1:2000), LBR (Proteintech, 12,398–1‐AP, 1:200), KAP1 (Proteintech, 66,630–1‐Ig, 1:200), LAP2 (Proteintech, 14,651–1‐Ap, 1:200). Cells were incubated at room temperature with the appropriate secondary antibodies, which included goat anti‐rabbit IgG H&L (Alexa Fluor 488, Abcam, ab150077, 1:200), goat anti‐rabbit IgG H&L (Alexa Fluor 647, Abcam, ab150079, 1:200), goat anti‐mouse IgG H&L (Alexa Fluor 488, Abcam, ab150113, 1:200), and goat anti‐mouse IgG H&L (Alexa Fluor 647, Abcam, ab150115, 1:200). Nuclei were stained with DAPI (Beyotime, C1006) or ProLong Diamond Antifade mountant with DAPI (Invitrogen, P36966) on slides (CITOTEST, 188105W). Imaging was performed using a Zeiss LSM 800 confocal laser scanning microscope (ZEISS, LSM800).

### 4.14 Trilineage Differentiation

We utilized human umbilical cord MSC osteogenic differentiation kits (Cyagen Biosciences, HUXUC‐90021) for osteogenic differentiation, human umbilical cord MSC adipogenic differentiation kits (Cyagen Biosciences, HUXUC‐90031) for adipogenic differentiation, and human umbilical cord MSC (Cyagen Biosciences, HUXUC‐90041) for chondrogenic differentiation. Trilineage differentiation medium was prepared in advance, and the protocols provided in the kits were followed for cell seeding and medium replacement. At the conclusion of osteogenic differentiation, cells were stained with Alizarin red, and at the conclusion of adipogenic differentiation, the cells were stained with Oil Red O. Following chondrogenic differentiation, the cells were paraffin‐embedded, sectioned, and subsequently stained with Alcian blue. Imaging was conducted using a ZEISS Observer A1 (ZEISS, Axio Observer A1) and ZEISS Imager A2 (ZEISS, Axio Observer A2).

### 4.15 Cell Cycle and Apoptosis Assay

A Cell Cycle and Apoptosis Assay Kit (Beyotime, C1052) was utilized for quantitation of apoptosis. Cells were fixed by addition of pre‐cooled 70% ethanol and subsequently stored in an ice incubation box at 4°C for a duration of 16 h. Following fixation, the cells were washed three times with PBS. Propidium iodide staining solution was then added to each tube containing the cell samples, and the cell precipitates were gently and thoroughly resuspended. Samples were incubated for 30 min at 37°C in the absence of light, after which they were washed three additional times with PBS. Testing was conducted using a BDC6 instrument.

### 4.16 Mitochondrial Membrane Potential Assay

We utilized a Mito‐Tracker Red CMXRos assay kit (Beyotime, C1049B) to assess mitochondrial membrane potential. A working solution of Mito‐Tracker Red CMXRos was prepared at a concentration of 40 μM. The medium and staining working solution were combined to achieve a final concentration of 160 nM. Samples were incubated in an incubator at 37°C for 30 min, followed by washing with PBS and fixation using 4% paraformaldehyde. Subsequently, DAPI was employed for nuclear staining. Imaging was conducted using a ZEISS Observer A1 (ZEISS, Axio Observer A1).

### 4.17 siRNA Transfection

siNC (human): 5′‐UUCUCCGAACGUGUCACGUTT‐3′, siTOP2A (human): 5′‐CCACGAAUAACCAUAGAAATT‐3′, siLMNB1 (human): 5′‐GAAGGAAUCUGAUCUUAAUTT‐3′, siLMNB2 (human): 5′‐GCGAGGUGAGUGGCAUCAATT‐3′, siLMNA (human): 5′‐CUGGACUUCCAGAAGAACATT‐3′, siSUV39H1 (human): 5′‐CGUGGAUUGUCUCAGGGAATT‐3′, siCBX5 (human): 5′‐GCAGAGCAAUGAUAUCGCUTT‐3′ purchased from GenePharma (Shanghai, China). A total of 80 nM of siRNA was used to transfect cells seeded at a density of 3000 cells/cm^2^ in a 6‐well plate using Lipofectamine 3000 (ThermoFisher, L3000015) to knock down the expression of *TOP2A* , *LMNB1* , *LMNB2* , *LMNA* , *SUV39H1* , and *CBX5* . At 72 h post‐transfection, proteins were extracted for Western blot analysis.

### 4.18 Gene Overexpression Cell Constructs

The empty vector plasmid (FV115 ZsGreen puro CON) and the overexpression plasmid (hTOP2A 3FLAG OE) were constructed by a company (FUBIO, Suzhou, Jiangsu Prov). The 293T‐FV115 ZsGreen puro CON (293T‐EV) and 293T‐hTOP2A 3FLAG OE (293T‐OE) cell lines were successfully established using the PEI (Sigma, 408727) transduction method (Yang et al. 2016). pLV3‐CMV‐MCS‐3FLAG‐CopGFP‐Puro (MiaoLingBio, P40122), pLV3‐CMV‐MCS‐3xFLAG‐mCherry‐Puro (MiaoLingBio, P54725), pLentiCRISPRV2‐U6‐TOP2A (human)‐sgRNA1‐Cas9‐Puro (MiaoLingBio, P33832), pLV3‐CMV‐LMNB1(human)‐3xFLAG‐mCherry‐Puro (MiaoLingBio, G88576), pLV3‐CMV‐LMNB2 (human)‐3xHA‐mCherry‐Puro (MiaoLingBio, P54507), pLV3‐CMV‐LMNA (human)‐mCherry‐Puro (MiaoLingBio, P49238), pLV3‐CMV‐SUV39H1 (human)‐3xFLAG‐Puro (MiaoLingBio, P47864), pLV3‐CMV‐CBX5 (human)‐3xFLAG‐CopGFP‐Puro (MiaoLingBio, P61047) were constructed using the same transduction method. Concurrently, MSCs were transduced with lentiviral supernatants, and the transduction efficiency was evaluated through drug screening and fluorescence analysis.

### 4.19 RNA‐Seq

RNA‐seq data were aligned using the Hisat2 algorithm (version 2.2.1) against the hg38 human genome. The featureCounts software (version 2.0.1) was employed to estimate read counts. Raw counts were utilized to assess the significance of differential expression between any two experimental groups, using the DESeq2 package. Gene expression changes were deemed significant if the *p* < 0.05. Gene set enrichment analysis was conducted using the clusterProfiler package (version 4.9.2) and the GseaVis package (version 0.0.9). All heatmaps were generated using the pheatmap package (version 1.0.12).

### 4.20 ATAC‐Seq

All samples were library constructed using the Hyperactive ATAC‐Seq Library Prep Kit for Illumina (Vazyme, TD711). After the library construction was completed, the samples were sent to company (Novogene, Beijing) for quality inspection, and sequencing was performed after qualified quality inspection. All samples were sequenced using the NovaSeq platform (Illumina) to generate paired‐end reads of 150 bp. The raw reads from ATAC‐Seq were initially processed with fastp (version 0.20.0). During this processing step, filtered data were obtained by eliminating sequencing junctions, reads containing more than six ambiguous bases (N), and other low‐quality reads. Specifically, low‐quality reads were defined as those with a quality score of less than 15 for more than 40% of the bases in the read, as well as reads shorter than 18 bp after trimming. The filtered data were subsequently aligned to the hg38 version of the human genome using Bowtie2, and all unmapped reads, non‐uniquely mapped reads, and PCR duplicates were removed.

### 4.21 CUT&Tag

All samples were library constructed using the Hyperactive Universal CUT&Tag Assay Kit for Illumina Pro (Vazyme, TD904). IgG antibody (CST, 4970S) was used in the negative control group, and TOP2A antibody (Abcam, ab52934) was used in the experimental group. After the library construction was completed, the samples were sent to company (Novogene, Beijing) for quality inspection, and sequencing was performed after qualified quality inspection. All samples were sequenced using the NovaSeq platform (Illumina) to produce paired‐end reads of 150 bp. Raw reads generated from the CUT&Tag protocol were initially processed with fastp (version 0.20.0). During this processing step, filtered data were obtained by eliminating sequencing junctions, reads containing more than six ambiguous bases N, and other low‐quality reads. Specifically, low‐quality reads were defined as those with a quality score of less than 20 for more than 40% of their bases, as well as reads shorter than 18 base pairs following trimming. The filtered data were then aligned to the hg38 version of the human genome and the Drosophila genome using Bowtie2. Subsequently, all unmapped reads, non‐uniquely mapped reads, and PCR duplicates were removed. For data analysis, a scale factor was initially determined based on the number of reads aligned to the Spike‐In genome. This scale factor was then applied to adjust the read depth, facilitate peak calling, and generate bigWig files for each sample, thereby correcting for variability among the samples.

### 4.22 CUT&Tag qPCR

Sequences of the promoter regions of TOP2A‐targeted genes were obtained by CUT&Tag sequencing analysis. Subsequently, we exported these sequences and designed primers using the NCBI database, which were then validated by quantitative PCR (qPCR) in complementary DNAs (cDNAs) derived from the CUT&Tag library.

### 4.23 Dual Luciferase Reporter Gene Assay

We utilized 293T‐FV115 ZsGreen puro CON (293T‐EV) and 293T‐hTOP2A 3FLAG OE (293T‐OE) cells and a dual luciferase reporter gene test kit (Beyotime, RG088S). We first performed the firefly luciferase assay, followed by the Renilla luciferase assay. Assays were conducted using the GloMax Discover System (Promega, GloMax Navigator). Renilla luciferase served as the internal reference, with the relative light unit (RLU) values obtained from the firefly luciferase assay divided by the RLU values obtained from the Renilla luciferase assay. Based on the calculated ratios, the degree of activation of the target reporter gene was compared across different samples.

### 4.24 Animal Behavioral Experiments and Analysis

We conducted open‐field experiments using 20‐week‐old mice and aging mice treated with TOP2Ai (PluriSIn #2). The specifications of the open field apparatus were 40 cm × 40 cm × 40 cm. It was essential to clean the open field device with alcohol both before and after each experiment. Mice were placed in the open field to record their movement trajectories. For the new object recognition experiments, which also utilized a 40 cm × 40 cm × 40 cm apparatus, we first selected an object for training the mice. During the formal experiment, both a new object and an old object were introduced into the device, and the movement trajectories of the mice were recorded. The Y‐maze spontaneous alternation experiments were conducted in a Y‐maze measuring 50 cm × 18 cm × 35 cm, with an angle of 120°. These experiments required counting the number of self‐alternations as well as the frequency of self‐alternations. We employed VisuTrack software (Shanghai XinSoft Information Technology Co.) to analyze the trajectories of the mice.

### 4.25 Statistics

We used GraphPad Prism 8.0.2 for plotting. Statistical significance was determined by the independent samples *t*‐test or the One‐way ANOVA. Data are presented as the mean ± SD using IBM SPSS Statistic 27, *p < 0.05, **p < 0.01, ***p < 0.001. The number of samples was indicated in each figure and figure legends. All in vitro experiments were replicated at least three times.

## Author Contributions

X.Z., P.S., and J.Z. supervised the study. K.C. performed most of the experiments. Y.W., performed the bioinformatics analyses. Y.P. performed in the screening aging biomarkers. D.Y., W.Z., Q.Z., Z.Z., R.M., C.X., A.H., Y.M., Y.X., Q.X. participated in partial experiments.

## Funding

This work was supported by the National Natural Science Foundation of China (81571994 to P.S., 81870432 and 81570567 to X.Z., 82372916 to J.Z.); Guangdong Basic and Applied Basic Research Foundation (2023A1515010449 and 2025A1515010366 to P.S., 2023A1515010660 and 2024A1515010341 to X.Z.); Natural Science Foundation of Guangdong Province (2024A1515140168 to J.Z.); and the Li Ka Shing Foundation (L1111 2008 to P. S.).

## Conflicts of Interest

The authors declare no conflicts of interest.

## Supporting information


**Figure S1:** Screening of senescence markers, isolation and identification of MSCs. Related to Figure 1. (A) UMAP identifying eight MSC subpopulations. (B) Dot heatmap showing the expression of MSC proliferation (*MKI67*, *PCNA*) and senescence indicators (*CDKN1A*, *SERPINE2*) in eight clusters. (C) WGCNA‐based network analysis revealed MSC senescence‐driving gene modules. Highlighting senescence‐associated gene modules (MEblue: *r* = −0.93, *p* = 2e‐10 and MEturquoise modules: *r* = 0.93, *p* = 5e‐10). (D) Phase‐contrast images of human umbilical cord‐derived MSCs at passages 0 (P0), 1 (P1), and 2 (P2). (E) Flow cytometry validation of MSC surface markers (CD73^+^/CD90^+^/CD105^+^/CD14^−^/CD34^−^/CD45^−^/HLA‐DR^−^). (F) Assessment of the trilineage differentiation potential of MSCs by Alizarin Red S (osteogenesis), Oil Red O (adipogenesis), and Alcian blue (chondrogenesis) staining. (D–F) Scale bar: 100 μm. Source data are available online for this figure.
**Figure S2:** Eplicative senescence correlates with TOP2A downregulation in MSCs. Related to Figure 1. (A) SA‐β‐gal staining (blue) of early passage and late passage MSCs. Scale bar: 50 μm. *N* = 5 biological repeats, Donor 2 (*p* < 0.001), Donor 3 (*p* < 0.001). (B) EdU staining (red) quantifying proliferative capacity of early passage and late passage MSCs. Scale bar: 50 μm. *N* = 4 biological repeats, Donor 2 (*p* < 0.001), Donor 3 (*p* < 0.001). (C) Colony formation assays of MSCs at early passage and late passage. *N* = 3 biological repeats, Donor 2 (*p* < 0.001), Donor 3 (*p* < 0.001). (D) Nuclear area quantification of early passage and late passage MSCs showing senescence‐associated hypertrophy. Scale bar: 10 μm. *N* = 200 cells per group, Donor 2 (*p* < 0.001), Donor 3 (*p* < 0.001). (E) Western blot analysis of TOP2A and p16 protein expression levels in replicative senescent MSCs. *N* = 3 biological repeats, TOP2A (*p =* 0.015), p16 (*p* = 0.004). (F) Densitometric quantification of western blot bands by ImageJ. *N* = 3 biological repeats, TOP2A (*p <* 0.001), p16 (*p* = 0.005). Early passage (EP) is defined as passage 4 (P4). Late passage (LP) is defined as P13. Statistical significance was determined using the independent samples *t*‐test. Data are presented as the mean ± SD. *p < 0.05, **p < 0.01, ***p < 0.001. Source data are available online for this figure.
**Figure S3:** Conserved age‐related TOP2A downregulation across multi‐omics databases. Related to Figure 1. (A) Expression levels of TOP2A and senescence‐related markers (*CDKN1A*, *CDKN2A*) in adipose‐derived MSCs from the GEO dataset GSE98440 (young vs. senescent). *N* = 4 biological repeats, *CDKN1A* (*p* = 0.017), *CDKN2A* (*p* = 0.105), *MKI67* (*p* = 0.016), *TOP2A* (*p* = 0.010). (B) Expression levels of TOP2A and senescence‐related markers (*CDKN1A*, *CDKN2A*) in bone marrow MSCs (BM‐MSCs) from GEO: GSE160273 (young vs. senescent). *CDKN1A* (*p* = 0.572), *CDKN2A* (*p* = 0.018), *MKI67* (*p* = 0.014), *TOP2A* (*p* = 0.011). (C) Age‐associated decline of TOP2A expression in multi‐human tissues in the ADEIP database. Statistical significance was determined the independent samples *t*‐test. Data are presented as the mean ± SD. *p < 0.05, **p < 0.01, ***p < 0.001. Source data are available online for this figure.
**Figure S4:** Multidimensional profiling of TOP2A knockdown effects on MSC senescence. Related to Figure 2. (A) SA‐β‐gal staining showing MSCs (blue). Scale bar: 50 μm. *N* = 8 biological repeats, *p* < 0.001. (B) MSC colony formation. *N* = 3 independent experiments, *p* < 0.001. (C) RT‐qPCR validation of *TOP2B* expression 24 h post‐TOP2Ai treatment in MSCs. *N* = 4 biological repeats, *CDKN1A* (*p =* 0.009), *CDKN2A* (*p =* 0.728), *TOP2A* (*p =* 0.038), *TOP2B* (*p =* 0.102), *MKI67* (*p* < 0.001). (D) Statistical analysis of staining Results for Osteoblasts (*p* < 0.001), Adipoblasts (*p =* 0.003), and Chondrocytes (*p* < 0.001), *N* = 3 biological repeats. (E) Heatmap illustrating SASP‐related factors (*IL6*, *IL1β*, and *MMP3*) upregulated in RNA‐seq data of TOP2Ai‐treated MSCs. (F) RT‐qPCR quantification of SASP factor mRNA levels (*p < 0.05 vs. DMSO). *N* = 3 biological repeats, *IL6* (*p =* 0.007), *IL1A* (*p* < 0.001), *IL1B* (*p =* 0.004), *MMP3* (*p =* 0.003), *AREG* (*p =* 0.005), *EREG* (*p* < 0.001), *ETS2* (*p =* 0.006), *CTSB* (*p =* 0.036), *PAPPA* (*p =* 0.0013), *SERPIEN1* (*p* < 0.001), *SERPIEN2* (*p* < 0.001). (G) GSEA showing suppression of mitochondrial genome maintenance pathways (NES = −1.51, *p* < 0.05). (H) Mito‐Tracker Red CMXRos staining revealing mitochondrial membrane potential depolarization in TOP2Ai‐treated MSCs. Scale bar: 50 μm. *N* = 200 cells per group, *p* < 0.001. (I) EdU incorporation assay (red) demonstrating proliferation inhibition in TOP2Ai‐treated MSCs. Scale bar: 100 μm. *N* = 10 biological repeats, *p* < 0.001. (J) Cell cycle distribution analyzed by PI staining: DMSO control vs. TOP2Ai‐treated MSCs. (K) Quantification of cell cycle phases. *N* = 3 independent experiments, G1 (*p =* 0.022), S (*p =* 0.150), G2 (*p =* 0.678). Statistical analysis significance determined by the independent samples *t*‐test. Data are presented as the mean ± SD. *p < 0.05, **p < 0.01, ***p < 0.001. Source data are available online for this figure.
**Figure S5:** Multi‐omics validation of TOP2A‐associated candidate genes. Related to Figure 4.
**Figure S6:** Functional rescue of mitochondrial and proliferative capacities by TOP2A overexpression. Related to Figure 6. (A) Mito‐Tracker Red CMXRos staining showing restored mitochondrial membrane potential in TOP2A‐overexpressing MSCs (MSC‐OE vs. MSC‐EV control). Scale bar: 50 μm. *N* = 200 cells per group, *p* < 0.001. (B) EdU incorporation assay (red) demonstrating recovered proliferation in the MSC‐OE group. Scale bar: 50 μm. *N* = 3 biological repeats, *p* < 0.001. Statistical significance determined by the independent samples *t*‐test. Data are presented as the mean ± SD. *p < 0.05, **p < 0.01, ***p < 0.001. Source data are available online for this figure.
**Figure S7:** Statistical analysis of SA‐β‐gal staining in the hippocampal regions and cortical layers. Related to Figure 7. (A, B) Statistical analysis of SA‐β‐gal staining in the hippocampal regions and cortical layers of the brains of naturally aging mice. *N* = 3 biological repeats, (A, *p* < 0.001, B, *p* < 0.001). (C, D) Statistical analysis of SA‐β‐gal staining in the hippocampal regions and cortical layers of brains of TOP2Ai‐treated mice. *N* = 3 biological repeats, (C, *p* < 0.001, D, *p* < 0.001). Statistical significance determined by the independent samples *t*‐test. Data are presented as the mean ± SD. *p < 0.05, **p < 0.01, ***p < 0.001. Source data are available online for this figure.
**Figure S8:** Other aging signs in TOP2Ai‐induced aging mice. Related to Figure 7. (A–C) Western blot analysis of TOP2A, Lamin B1, and heterochromatin‐associated markers in mesoderm‐derived tissues (Adipose, Heart, Kidney). *N* = 3 biological repeats, (A, Adipose, *N* = 3 biological repeats, TOP2A (*p* = 0.037), Lamin B1 (*p* = 0.022), H3K9me3 (*p* < 0.001). B, Heart, *N* = 3 biological repeats, TOP2A (*p* = 0.027), Lamin B1 (*p* = 0.049), KAP1 (*p* = 0.089). C, Kidney, *N* = 4 biological repeats, TOP2A (*p* = 0.169), Lamin B1 (*p* = 0.015), KAP1 (*p* = 0.058), H3K9me3 (*p* = 0.002), p21 (*p* = 0.086), p16 (*p =* 0.002). (D) Western blot analysis of TOP2A, Lamin B1, and heterochromatin‐associated markers in tissues derived from other germ layers (skin), *N* = 4 biological repeats, TOP2A (*p* = 0.003), Lamin B1 (*p* = 0.002), KAP1 (*p* = 0.080), H3K9me3 (*p* = 0.148), p16 (*p* < 0.001). Statistical significance determined by the independent samples *t*‐test. Data are presented as the mean ± SD. *p < 0.05, **p < 0.01, ***p < 0.001. Source data are available online for this figure.

## Data Availability

The data that supports the findings of this study are available in the Supporting Information of this article. The data that support the findings of this study are available in Genome Sequence Archive, GSA at https://ngdc.cncb.ac.cn/gsa‐human, number HRA012013 (CUT&Tag), HRA012014 (ATAC‐Seq), HRA012015 (RNA‐Seq).
