# Supplementary material for: TOP2A Maintains Heterochromatin Stability by Sustaining LMNB1 Expression to Counteract Senescence
Source: Aging Cell. 2026 Nov 2;25(11):e70787. doi: 10.1111/acel.70787 (PMC13727716; doi:10.1111/acel.70787)
Supplement: Supplementary file 1 [file ACEL-25-e70787-s001.docx]

**Supplemental information**

**TOP2A maintains heterochromatin stability by sustaining LMNB1 expression to counteract senescence**

Ke Cheng, Yaru Wang, Yueyuan Pan, Wenjun Zhang, Denghong Yu, Qi Zhou, Zhiqian Zhong, Ruyi Mei, Chaoliang Xu, Ayidana Hayierhan, Yumei Ma, Yien Xu, Qingdong Xie, Pingnan Sun Junzhi Zhou, Xiaoling Zhou

**Figure S1. Screening of senescence markers, isolation and identification of MSCs. Related to Figure 1.** (**A**) UMAP identifying eight MSC subpopulations. (**B**) Dot heatmap showing the expression of MSC proliferation (*MKI67*, *PCNA*) and senescence indicators (*CDKN1A*, *SERPINE2*) in eight clusters. (**C**) WGCNA-based network analysis revealed MSC senescence-driving gene modules. highlighting senescence-associated gene modules (MEblue: r=-0.93, *p*=2e-10 and MEturquoise modules: r=0.93, *p*=5e-10). (**D**) Phase-contrast images of human umbilical cord-derived MSCs at passages 0 (P0), 1 (P1), and 2 (P2). (**E**) Flow cytometry validation of MSC surface markers (CD73^+^/CD90^+^/CD105^+^/CD14^-^/CD34^-^/CD45^-^/HLA-DR^-^). (**F**) Assessment of the trilineage differentiation potential of MSCs by Alizarin Red S (osteogenesis), Oil Red O (adipogenesis), and Alcian blue (chondrogenesis) staining. (**D**-**F**) Scale bar: 100 μm. Source data are available online for this figure.

**Figure S2. eplicative senescence correlates with TOP2A downregulation in MSCs. Related to Figure 1.** (**A**) SA-β-gal staining (blue) of early passage and late passage MSCs. Scale bar: 50 μm. N = 5 biological repeats, Donor 2 (*p* < 0.001), Donor 3 (*p* < 0.001). (**B**) EdU staining (red) quantifying proliferative capacity of early passage and late passage MSCs. Scale bar: 50 μm. N = 4 biological repeats, Donor 2 (*p* < 0.001), Donor 3 (*p* < 0.001). (**C**) Colony formation assays of MSCs at early passage and late passage. N = 3 biological repeats, Donor 2 (*p* < 0.001), Donor 3 (*p* < 0.001). (**D**) Nuclear area quantification of early passage and late passage MSCs showing senescence-associated hypertrophy. Scale bar: 10 μm. N = 200 cells per group, Donor 2 (*p* < 0.001), Donor 3 (*p* < 0.001). (**E**) Western blot analysis of TOP2A and p16 protein expression levels in replicative senescent MSCs. N = 3 biological repeats, TOP2A (*p =* 0.015), p16 (*p* = 0.004). (**F**) Densitometric quantification of western blot bands by ImageJ. N = 3 biological repeats, TOP2A (*p <* 0.001), p16 (*p* = 0.005). Early passage (EP) is defined as passage 4 (P4). Late passage (LP) is defined as P13. Statistical significance was determined using the independent samples *t*-test. Data are presented as the mean ±SD. **p* < 0.05, ***p* < 0.01, ****p* < 0.001. Source data are available online for this figure.

**Figure S3. Conserved age-related TOP2A downregulation across multi-omics databases. Related to Figure 1.** (**A**) Expression levels of TOP2A and senescence-related markers (*CDKN1A*, *CDKN2A*) in adipose-derived MSCs from the GEO dataset GSE98440 (young vs. senescent). N = 4 biological repeats, *CDKN1A* (*p* = 0.017), *CDKN2A* (*p* = 0.105), *MKI67* (*p* = 0.016), *TOP2A* (*p* = 0.010). (**B)** Expression levels of TOP2A and senescence-related markers (*CDKN1A*, *CDKN2A*) in bone marrow MSCs (BM-MSCs) from GEO: GSE160273 (young vs. senescent). *CDKN1A* (*p* = 0.572), *CDKN2A* (*p* = 0.018), *MKI67* (*p* = 0.014), *TOP2A* (*p* = 0.011). (**C**) Age-associated decline of TOP2A expression in multi-human tissues in the ADEIP database. Statistical significance was determined the independent samples *t*-test. Data are presented as the mean ±SD. **p* < 0.05, ***p* < 0.01, ****p* < 0.001. Source data are available online for this figure.

**Figure S4. Multidimensional profiling of TOP2A knockdown effects on MSC senescence. Related to Figure 2.** (**A**) SA-β-gal staining showing MSCs (blue). Scale bar: 50 μm. N = 8 biological repeats, *p* < 0.001. (**B**) MSC colony formation. N = 3 independent experiments, *p* < 0.001. (**C**) RT-qPCR validation of *TOP2B* expression 24 h post-TOP2Ai treatment in MSCs. N = 4 biological repeats, *CDKN1A* (*p =* 0.009), *CDKN2A* (*p =* 0.728), *TOP2A* (*p =* 0.038), *TOP2B* (*p =* 0.102), *MKI67* (*p* < 0.001). (**D**) Statistical analysis of staining Results for Osteoblasts (*p* < 0.001), Adipoblasts (*p =* 0.003), and Chondrocytes (*p* < 0.001), N = 3 biological repeats. (**E**) Heatmap illustrating SASP-related factors (*IL6*, *IL1β*, and *MMP3*) upregulated in RNA-seq data of TOP2Ai-treated MSCs. (**F**) RT-qPCR quantification of SASP factor mRNA levels (**p*<0.05 vs. DMSO). N = 3 biological repeats, *IL6* (*p =* 0.007), *IL1A* (*p* < 0.001), *IL1B* (*p =* 0.004), *MMP3* (*p =* 0.003), *AREG* (*p =* 0.005), *EREG* (*p* < 0.001), *ETS2* (*p =* 0.006), *CTSB* (*p =* 0.036), *PAPPA* (*p =* 0.0013), *SERPIEN1* (*p* < 0.001), *SERPIEN2* (*p* < 0.001). (**G**) GSEA showing suppression of mitochondrial genome maintenance pathways (NES=-1.51, *p*<0.05). (**H**) Mito-Tracker Red CMXRos staining revealing mitochondrial membrane potential depolarization in TOP2Ai-treated MSCs. Scale bar: 50 μm. N = 200 cells per group, *p* < 0.001. (**I**) EdU incorporation assay (red) demonstrating proliferation inhibition in TOP2Ai-treated MSCs. Scale bar: 100 μm. N = 10 biological repeats, *p* < 0.001. (**J**) Cell cycle distribution analyzed by PI staining: DMSO control vs. TOP2Ai-treated MSCs. (**K**) Quantification of cell cycle phases. N = 3 independent experiments, G1 (*p =* 0.022), S (*p =* 0.150), G2 (*p =* 0.678). Statistical analysis significance determined by the independent samples *t*-test. Data are presented as the mean ±SD. **p* < 0.05, ***p* < 0.01, ****p* < 0.001. Source data are available online for this figure.

Figure S5. Multi-omics validation of TOP2A-associated candidate genes. Related to Figure 4.

(**A**) ATAC-seq tracks showing chromatin accessibility changes at *DMC1*, *DEPDC1*, *CLSPN*, and *MKI67* loci. (**B**) RNA-seq expression profiles of *DMC1*, *DEPDC1*, *CLSPN*, and *MKI67*. N = 3 biological repeats, *DMC1* (*p* < 0.001), *DEPDC1* (*p* < 0.001), *CLSPN* (*p* < 0.001), *MKI67* (*p* < 0.001). (**C**) CUT&Tag binding profiles at *DMC1*, *DEPDC1*, and *CLSPN* loci (*MKI67* binding signal not detected). N = 3 biological repeats, *DMC1* (*p =* 0.025), *DEPDC1* (*p =* 0.003), *CLSPN* (*p* < 0.001). Statistical significance determined by the independent samples *t*-test. Data are presented as the mean ±SD. **p* < 0.05, ***p* < 0.01, ****p* < 0.001. Source data are available online for this figure.

**Figure S6. Functional rescue of mitochondrial and proliferative capacities by TOP2A overexpression. Related to Figure 6.** (**A**) Mito-Tracker Red CMXRos staining showing restored mitochondrial membrane potential in TOP2A-overexpressing MSCs (MSC-OE vs. MSC-EV control). Scale bar: 50 μm. N = 200 cells per group, *p* < 0.001. (**B**) EdU incorporation assay (red) demonstrating recovered proliferation in the MSC-OE group. Scale bar: 50 μm. N = 3 biological repeats, *p* < 0.001. Statistical significance determined by the independent samples *t*-test. Data are presented as the mean ±SD. **p* < 0.05, ***p* < 0.01, ****p* < 0.001. Source data are available online for this figure.

##### Figure S7. Statistical analysis of SA-β-gal staining in the hippocampal regions and cortical layers. Related to Figure 7. (A,B) Statistical analysis of SA-β-gal staining in the hippocampal regions and cortical layers of the brains of naturally aging mice. N = 3 biological repeats, (A, *p* < 0.001, B, *p* < 0.001). (C,D) Statistical analysis of SA-β-gal staining in the hippocampal regions and cortical layers of brains of TOP2Ai-treated mice. N = 3 biological repeats, (C, *p* < 0.001, D, *p* < 0.001). Statistical significance determined by the independent samples *t*-test. Data are presented as the mean ±SD. **p* < 0.05, ***p* < 0.01, ****p* < 0.001. Source data are available online for this figure.

**Figure S8. Other aging signs in TOP2Ai-induced aging mice. Related to Figure** 7. (**A-C**) Western blot analysis of TOP2A, Lamin B1, and heterochromatin-associated markers in mesoderm-derived tissues (Adipose, Heart, Kidney). N = 3 biological repeats, (**A**, Adipose, N = 3 biological repeats, TOP2A (*p* = 0.037), Lamin B1 (*p* = 0.022), H3K9me3 (*p* < 0.001). **B**, Heart, N = 3 biological repeats, TOP2A (*p* = 0.027), Lamin B1 (*p* = 0.049), KAP1 (*p* = 0.089). **C**, Kidney, N = 4 biological repeats, TOP2A (*p* = 0.169), Lamin B1 (*p* = 0.015), KAP1 (*p* = 0.058), H3K9me3 (*p* = 0.002), p21 (*p* = 0.086), p16 (*p =* 0.002). (**D**) Western blot analysis of TOP2A, Lamin B1, and heterochromatin-associated markers in tissues derived from other germ layers (skin), N = 4 biological repeats, TOP2A (*p* = 0.003), Lamin B1 (*p* = 0.002), KAP1 (*p* = 0.080), H3K9me3 (*p* = 0.148), p16 (*p* < 0.001). Statistical significance determined by the independent samples *t*-test. Data are presented as the mean ±SD. **p* < 0.05, ***p* < 0.01, ****p* < 0.001. Source data are available online for this figure.
